# Noninvasive prenatal testing of α-thalassemia and β-thalassemia through population-based parental haplotyping

**DOI:** 10.1186/s13073-021-00836-8

**Published:** 2021-02-05

**Authors:** Chao Chen, Ru Li, Jun Sun, Yaping Zhu, Lu Jiang, Jian Li, Fang Fu, Junhui Wan, Fengyu Guo, Xiaoying An, Yaoshen Wang, Linlin Fan, Yan Sun, Xiaosen Guo, Sumin Zhao, Wanyang Wang, Fanwei Zeng, Yun Yang, Peixiang Ni, Yi Ding, Bixia Xiang, Zhiyu Peng, Can Liao

**Affiliations:** 1 BGI Genomics, BGI-Shenzhen, Shenzhen, 518083 China; 2 Tianjin Medical Laboratory, BGI-Tianjin, BGI-Shenzhen, Tianjin, 300308 China; 3 Department of Prenatal Diagnostic Center, Guangzhou Women and Children’s Medical Center, Guangzhou Medical University, Guangzhou, 510623 China; 4 BGI-Wuhan Clinical Laboratories, BGI-Shenzhen, Wuhan, 490079 China; 5 Department of Obstetrics and Gynecology, The Second Affiliated Hospital of Zhengzhou University, Zhengzhou, 450052 China

**Keywords:** NIPT, Recessive monogenic diseases, Haplotypes, Population-based haplotyping, α-Thalassemia, β-Thalassemia

## Abstract

**Background:**

Noninvasive prenatal testing (NIPT) of recessive monogenic diseases depends heavily on knowing the correct parental haplotypes. However, the currently used family-based haplotyping method requires pedigrees, and molecular haplotyping is highly challenging due to its high cost, long turnaround time, and complexity. Here, we proposed a new two-step approach, population-based haplotyping-NIPT (PBH-NIPT), using α-thalassemia and β-thalassemia as prototypes.

**Methods:**

First, we deduced parental haplotypes with Beagle 4.0 with training on a large retrospective carrier screening dataset (4356 thalassemia carrier screening-positive cases). Second, we inferred fetal haplotypes using a parental haplotype-assisted hidden Markov model (HMM) and the Viterbi algorithm.

**Results:**

With this approach, we enrolled 59 couples at risk of having a fetus with thalassemia and successfully inferred 94.1% (111/118) of fetal alleles. We confirmed these alleles by invasive prenatal diagnosis, with 99.1% (110/111) accuracy (95% CI, 95.1–100%).

**Conclusions:**

These results demonstrate that PBH-NIPT is a sensitive, fast, and inexpensive strategy for NIPT of thalassemia.

**Supplementary Information:**

The online version contains supplementary material available at 10.1186/s13073-021-00836-8.

## Background

The discovery of cell-free fetal DNA enables noninvasive prenatal testing (NIPT) for common aneuploidies [1, 2], microdeletion/microduplication syndromes [3–5], and monogenic disorders. Initially, NIPT of monogenic disorders focused on detecting de novo or paternally inherited variants responsible for dominant monogenic disorders [6, 7]. Reports indicate that the average genomic carrier burden for severe pediatric recessive variants is 2.8 per person [8] and that the cumulative prevalence among live births is approximately 0.8% [9]. NIPT for most recessive monogenic disorders involves several technical challenges and has only been made clinically available for a limited number of recessive conditions [10] despite the relatively high prevalence of such disorders, because analysis of maternally inherited fetal alleles has been hampered by the high background of maternal DNA in cell-free DNA (cfDNA) [11]. The current approaches for NIPT of recessive diseases are typically classified into two categories [12, 13]: relative mutation dosage (RMD) analysis [14] and relative haplotype dosage (RHDO) analysis [15]. The RMD approach focuses on quantitative comparisons between variant and wild-type alleles present in cfDNA and has relatively high sensitivity and specificity [14, 16]. This approach is powerful for detecting single nucleotide variants (SNVs) and small insertions/deletions (InDels) but usually cannot detect large InDels and copy number variants (CNVs) [17–19]. Its performance is also affected by sequencing errors and amplification bias of low-abundance fetal variants in cfDNA [20]. Unlike the RMD approach, the RHDO approach determines the relative proportions of variant and normal haplotypes in maternal plasma [21] and can theoretically detect most types of variants, including large InDels and CNVs, in one test [16, 22]. However, RHDO analysis requires parental haplotype information [23]. Although molecular phasing approaches to determine parental haplotypes, including linked-read sequencing [24, 25] and targeted locus amplification (TLA) [26], have not been widely used in clinical settings due to their high cost and complex procedures [27–32], population-based parental haplotyping provides an alternative approach due to its rapid turnaround and inexpensive and relatively simple procedures. However, the use of this method has been limited to a founder variant (*GBA* gene, c.1226A>G) [33].

Thalassemia causes hemoglobin deficiency and affects approximately 4.4 per 10,000 live births worldwide [34]. Its genetic complexity involves three types of variants: SNVs, InDels, and CNVs. In southern China, the most prevalent variants of α-thalassemia are -α^3.7^ deletion, --^SEA^ deletion, -α^4.2^ deletion, *HBA2* c.369C>G, and *HBA2* c.427T>C, while those of β-thalassemia are *HBB* c.126_129delCTTT, *HBB* c.52A>T, *HBB* c.316-197C>T, *HBB* c.-78A>G, and *HBB* c.79G>A [35, 36]. Our population screening data for thalassemia [35] shows that these 10 variants account for 87.9% of β-thalassemia carriers and 96.5% of α-thalassemia carriers (Additional file 1: Fig. S1).

In the present study, we proposed a novel population-based haplotyping-NIPT method (PBH-NIPT) for α-thalassemia and β-thalassemia in which nonfounder variants were detected when the sample size of the reference panel (population data used to infer parental haplotypes) was sufficiently large for accurate deduction of parental haplotypes. The PBH-NIPT model was trained on a large retrospective carrier screening dataset, and its accuracy was verified via invasive prenatal diagnosis. In addition, we assessed the effect of the reference panel sample size on the outcomes of PBH-NIPT.

## Methods

### Patients and samples

The ethics committees of Guangzhou Women and Children’s Medical Center and BGI approved this study (approval numbers: 2017102408 and BGI-IRB 18043). Fifty-nine couples at risk of having a fetus with thalassemia provided written informed consent. The clinical features of the participants are provided in the supplement (Additional file 2: Table S1). We collected 5 ml of blood from each parent. We promptly isolated maternal plasma using a two-step centrifugation method [37]. We used 10 ml of amniotic fluid (AF) or 5 mg of chorionic villus sample (CVS) for invasive prenatal diagnosis.

### Sequencing library preparation

We extracted cfDNA from maternal plasma using a QIAamp Circulating Nucleic Acid Kit (Qiagen, Dusseldorf, Germany) and extracted parental gDNA from peripheral blood and fetal DNA from CVS or AF using a QIAamp DNA Mini Kit (Qiagen).

We used gDNA (500 ng) for library construction and fragmented it ultrasonically with a Bioruptor Pico (Diagenode, Liege, Belgium), yielding 300–700-bp fragments. We then performed end repair, phosphorylation, and A-tailing reactions on the sheared DNA and ligated BGISEQ adaptors with specific barcodes to the A-tailed products. We performed 4–6 cycles of polymerase chain reaction (PCR) amplification to enrich the target regions and performed hybridization capture according to the NimbleGen protocols after pooling twenty barcoded gDNA libraries in equal amounts. Finally, we performed circularization of the post-capture library to generate circular single-stranded DNA (ssDNA). We prepared the maternal plasma DNA library using the same method except without fragmentation and pooled eight cfDNA libraries in equal amounts. After quantitation using Qubit 3.0 (Thermo Fisher, Waltham, USA), we used rolling circle replication to form DNA nanoballs (DNBs) from the ssDNA and loaded each DNB into 1 lane to be processed for 100-bp paired-end sequencing on the BGISEQ-500 and MGISEQ-2000 platforms (BGI, Shenzhen, China).

### Reference panel construction

We generated the reference panel from 4356 thalassemia carrier screening-positive cases. Of the total 4356 cases, 3867 were obtained from our previously published paper [35], and 489 were obtained from unpublished in-house data.

We first used our previously published algorithm [35] to call SNPs from 4356 positive carriers and then filtered SNPs with a sequencing depth of less than 20-fold in more than 2% of the population or with an allele ratio between 5 and 40% in more than 70% of heterozygous individuals in the population. We used the publicly available software Beagle (version 4.0) to construct haplotypes for 4356 individuals and used these data as the reference panel for the next step. Since SNPs and InDels are the acceptable input for Beagle, we treated CNVs as SNPs in the phasing procedure. CNVs are represented as the VCF format of SNPs in the Beagle input file (VCF format), where the genomic position is the start position of the CNV, and the genotypes “0/1” and “1/1” represent heterozygous and homozygous CNVs, respectively.

### Construction of parental haplotypes by PBH

We aligned the sequence reads from parental gDNA and maternal plasma DNA to the reference human genome (hg19) using BWA version 0.7.12. We marked duplicate reads with Picard version 1.87 and performed variant calling as previously described [35]. We also treated CNVs as SNPs in the phasing procedure. We used the haplotypes of the reference panel and the genotypes of the parents as inputs to deduce parental haplotypes with Beagle 4.0. Finally, we used only heterozygous SNPs to represent parental haplotypes.

### NIPT of thalassemia

We calculated the fetal fraction (FF) as described in Additional file 3 and inferred fetal haplotypes inherited from the father and mother separately. First, we determined paternal inheritance using paternal informative SNPs, which were heterozygous in the father but homozygous in the mother. Second, we determined maternal inheritance using maternal informative SNPs, which included two types of SNPs: (1) SNPs heterozygous in the mother but homozygous in the father and (2) SNPs heterozygous in the parents in the blocks where the first step inferred the fetal inherited haplotype from the father. Because informative SNPs linked to the inherited haplotype are overrepresented in maternal plasma, we applied the hidden Markov model (HMM) and Viterbi algorithm [38] to determine the fetal genotypes of pathogenic sites (Additional file 3: Supplementary Methods). For samples with CNVs, all SNPs in the CNV region were not selected as informative SNPs to perform Viterbi decoding.

### Invasive prenatal diagnosis of thalassemia

We performed invasive prenatal diagnosis via chorionic villus sampling or amniocentesis in accordance with standard protocols. We determined fetal genotypes through gap-PCR and reverse dot blot PCR (RDB-PCR).

### The effect of the reference panel sample size on the outcomes of PBH-NIPT

To assess the effect of the reference panel sample size on the outcomes of PBH-NIPT, we randomly selected one-half, one-quarter, one-sixth, one-eighth, one-twelfth, and 50 of the samples from the total reference panel and performed three independent tests.

## Results

**Fig. 1:**
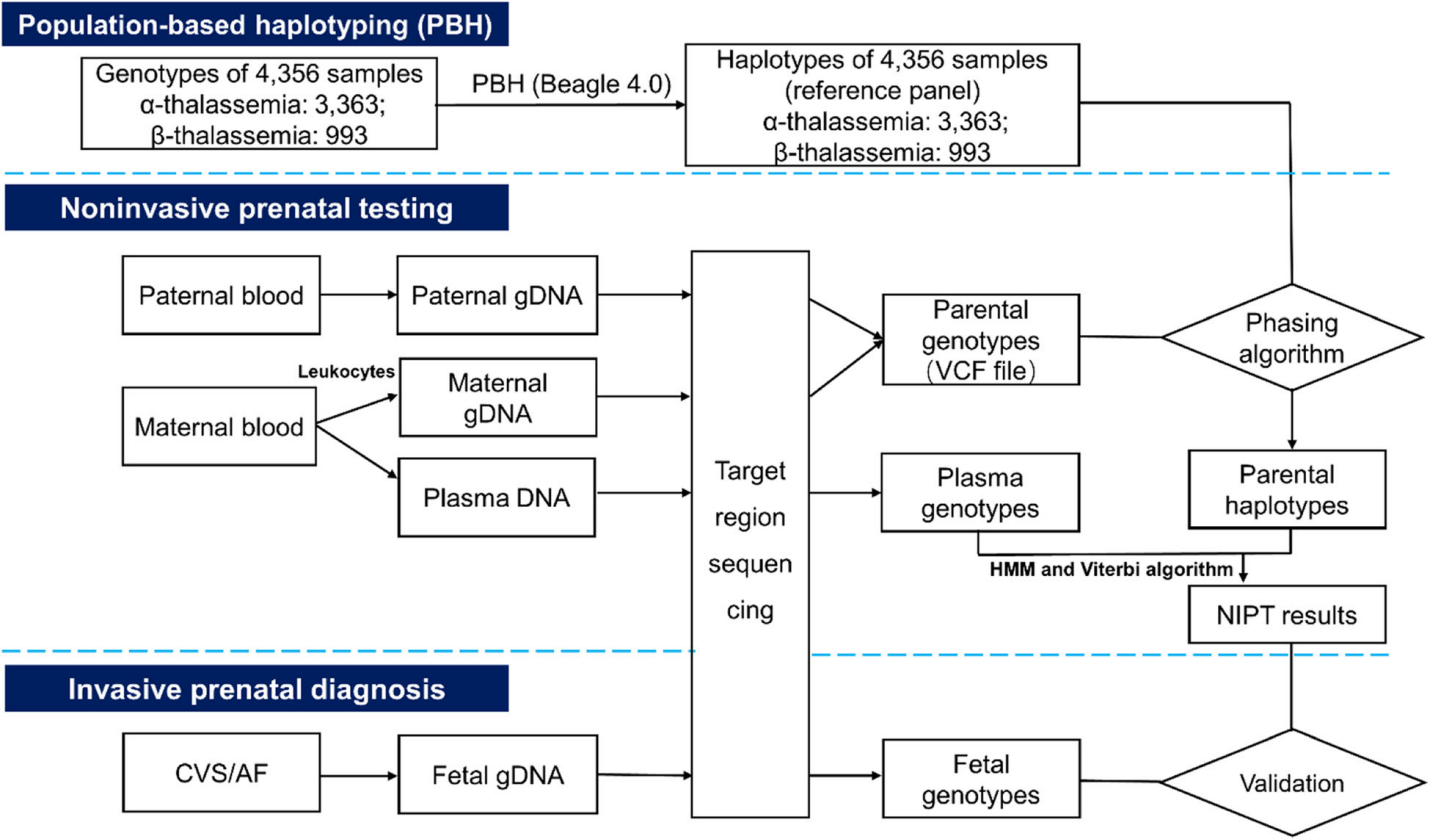
Population-based haplotyping-noninvasive prenatal testing (PBH-NIPT) workflow. The workflow involves 3 parts. We first constructed haplotypes of 59 couples using the haplotypes of 4356 samples as the reference panel. Then, we inferred fetal haplotypes using a hidden Markov model (HMM) and the Viterbi algorithm. Finally, we used invasive prenatal diagnosis results to validate PBH-NIPT results

As shown in Fig. 1, the PBH-NIPT workflow involves the following steps. First, we generated the reference panel from 4356 thalassemia carrier screening-positive cases. Of the total 4356 cases, 3867 were obtained from our previously published paper [35], and 489 were obtained from unpublished in-house data. Second, we enrolled 59 couples in whom both partners carried at least one of the 10 aforementioned variants and were at risk of having a fetus with thalassemia major or intermedia [39] (Additional file 2: Table S1). The average gestational age at the time of collection was 12.6^+3^ weeks (range 10^+1^–22 weeks), and the average FF was 15.4% (range 6.0–26.1%) (Additional file 4: Table S2). We subjected genomic DNA (gDNA) of the couples and fetuses as well as maternal cfDNA to hybridization-based capture and sequencing using a strategy previously described for thalassemia carrier screening [35]. We obtained an average target region coverage of 177-fold (range 56–678) in maternal plasma and 203-fold (range 85–360) in parental gDNA. Third, we inferred parental haplotypes by PBH (see the “Methods” section and Fig. 1). To evaluate the reliability of PBH, we also constructed parental haplotypes by family-based haplotyping (FBH) and calculated the percentage of concordant single-nucleotide polymorphisms (SNPs) phased by these two methods (Additional file 3: Supplementary Methods). The average concordance rates of phased SNPs in the maternal and paternal haplotypes were 98.7% (range 87.5–100%) and 95.7% (range 59.2–100%), respectively (Additional file 5: Fig. S2; Additional file 6: Table S3).

**Fig. 2:**
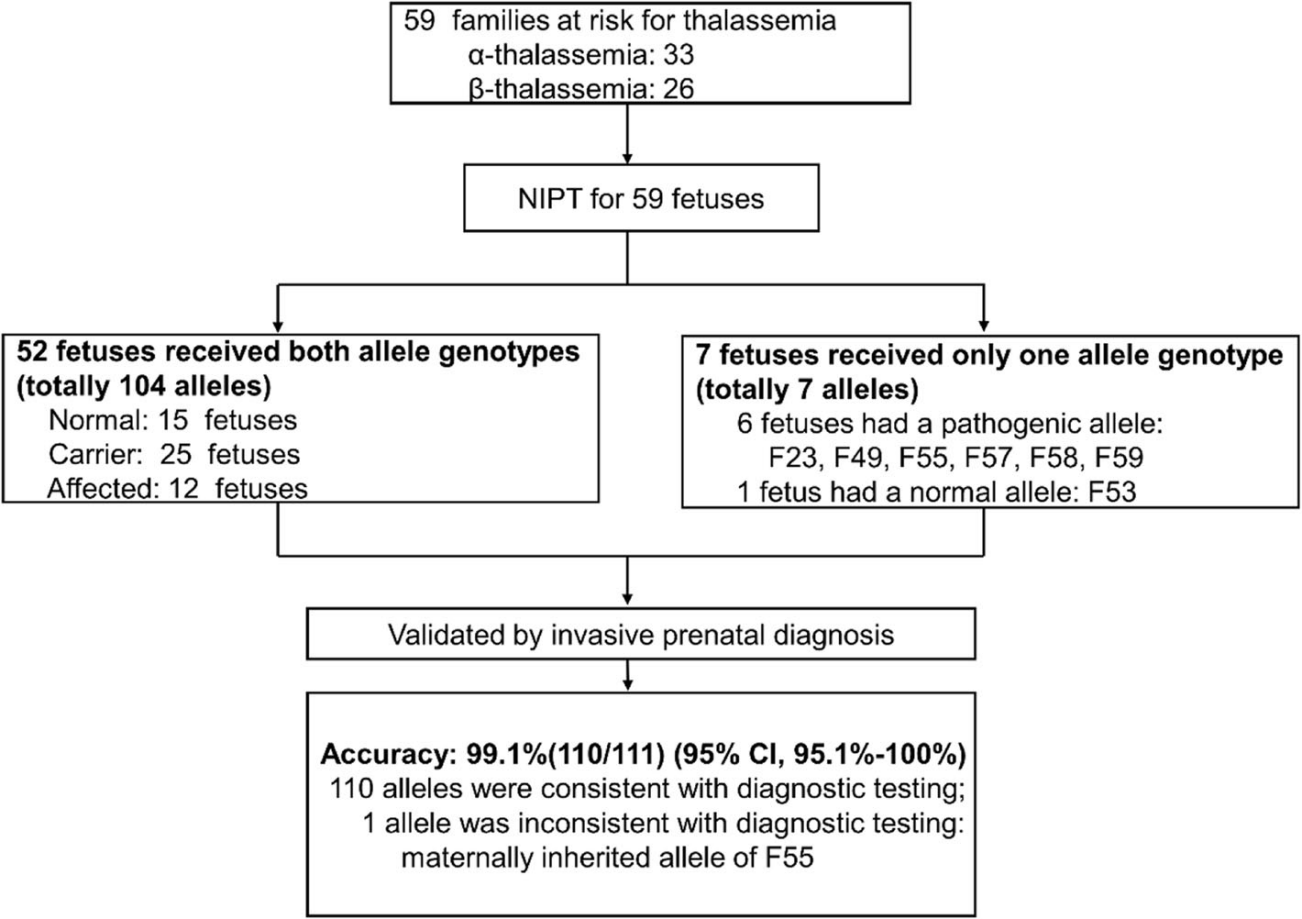
Outcomes of PBH-NIPT. We performed PBH-NIPT on 59 couples. Fifty-nine fetuses had 111 total alleles confirmed by invasive prenatal diagnosis, with 99.1% accuracy (95% CI, 95.1–100%). Fifty-two fetuses had both alleles detected for a total of 104 alleles; 7 fetuses had only one allele detected

To correctly infer fetal genotypes of pathogenic sites (rather than all SNPs), we developed a hidden Markov model (HMM) and used the Viterbi algorithm. We calculated a confidence score (CS), defined as the probability of obtaining the correct NIPT result, to evaluate the reliability of each prediction. A “no-call” condition was defined when (1) the CS was less than 0.99 or (2) the inferred haplotype contained two haplotype blocks (pathogenic and normal), and neither block spanned the target gene (*HBB* or *HBA*) (Additional file 3: Supplementary Methods). Accordingly, NIPT successfully inferred 111/118 (94.1%) alleles, and invasive prenatal diagnosis confirmed these alleles, with 99.1% (110/111 alleles) accuracy (95% CI, 95.1–100%) (Table 1, Fig. 2, and Additional file 7: Fig. S3). Among these 59 fetuses, 52 had both alleles detected; of these 52 fetuses, 15 were normal, 25 were carriers, and 12 were affected. Seven fetuses had only one allele successfully detected, and the other allele failed, with a CS of less than 0.99 (Table 1 and Fig. 2). Among the 7 fetuses with only one allele inferred by NIPT, 6 inherited the pathogenic allele. Obviously, invasive prenatal diagnosis was needed, which we used to clarify that 4 fetuses were affected and 2 were carriers.

**Table 1:** Noninvasive prenatal testing of thalassemia

Family	Gene	FF (%)	No. of maternal informative SNPs		No. of paternal informative SNPs		Mat Hap	Pat Hap	${\mathbf{\mathsf{CS}}}_{\mathbf{\mathsf{mat}}}$ (%)	${\mathbf{\mathsf{CS}}}_{\mathbf{\mathsf{pat}}}$ (%)	NIPT (Mat/Pat)	Invasive prenatal diagnosis (Mat/Pat)
			For M_**p**_	For M_**n**_	For P_**p**_	For P_**n**_						
**52 fetuses received both allele genotypes**												
F01	*HBB*	11.5	70	0	54	0	M_p_	P_p_	100	100	c.52A>T/c.126_129delCTTT	c.52A>T/c.126_129delCTTT
F02	*HBB*	9.4	0	99	0	35	M_n_	P_n_	100	100	N/N	N/N
F03	*HBB*	16.0	0	45	28	13	M_n_	P_n_	100	100	N/N	N/N
F04	*HBB*	12.4	40	0	57	0	M_p_	P_p_	100	100	c.126_129delCTTT/c.126_129delCTTT	c.126_129delCTTT/c.126_129delCTTT
F05	*HBB*	10.2	0	66	53	0	M_n_	P_p_	100	100	N/c.126_129delCTTT	N/c.126_129delCTTT
F06	*HBB*	15.9	36	0	0	92	M_p_	P_n_	100	100	c.126_129delCTTT/N	c.126_129delCTTT/N
F07	*HBB*	12.8	0	110	19	0	M_n_	P_p_	100	100	N/c.126_129delCTTT	N/c.126_129delCTTT
F08	*HBB*	21.8	43	31	33	40	M_p_	P_n_	100	100	c.126_129delCTTT/N	c.126_129delCTTT/N
F09	*HBB*	15.4	12	0	0	64	M_p_	P_n_	100	100	c.126_129delCTTT/N	c.126_129delCTTT/N
F10	*HBB*	17.6	26	3	0	25	M_p_	P_n_	100	100	c.126_129delCTTT/N	c.126_129delCTTT/N
F11	*HBB*	15.5	0	28	23	47	M_n_	P_n_	100	100	N/N	N/N
F12	*HBB*	7.0	0	105	0	21	M_n_	P_n_	100	100	N/N	N/N
F13	*HBB*	10.2	0	161	16	0	M_n_	P_p_	100	100	N/c.126_129delCTTT	N/c.126_129delCTTT
F14	*HBB*	15.1	60	0	70	0	M_p_	P_p_	100	100	c.126_129delCTTT/c.-78A>G	c.126_129delCTTT/c.-78A>G
F15	*HBB*	16.0	167	8	0	25	M_p_	P_n_	100	100	c.316-197C>T/N	c.316-197C>T/N
F16	*HBB*	6.0	85	0	0	51	M_p_	P_n_	100	100	c.316-197C>T/N	c.316-197C>T/N
F17	*HBB*	22.0	96	0	11	31	M_p_	P_n_	100	100	c.316-197C>T/N	c.316-197C>T/N
F18	*HBB*	15.5	72	0	3	42	M_p_	P_n_	100	100	c.-78A>G/N	c.-78A>G/N
F19	*HBB*	16.0	8	102	0	37	M_n_	P_n_	100	100	N/N	N/N
F20	*HBB*	13.5	0	59	0	4	M_n_	P_n_	100	100	N/N	N/N
F21	*HBB*	14.7	0	67	0	66	M_n_	P_n_	100	100	N/N	N/N
F22	*HBB*	14.5	38	0	39	0	M_p_	P_p_	100	100	c.52A>T/c.126_129delCTTT	c.52A>T/c.126_129delCTTT
F24	*HBB*	11.7	0	34	0	32	M_n_	P_n_	100	100	N/N	N/N
F25	*HBB*	13.1	0	40	0	61	M_n_	P_n_	100	100	N/N	N/N
F26	*HBB*	16.3	85	0	28	0	M_p_	P_p_	100	100	c.126_129delCTTT/c.-78A>G	c.126_129delCTTT/c.-78A>G
F27	*HBA*	16.0	21	0	25	0	M_p_	P_p_	100	100	- -^SEA^/- -^SEA^	- -^SEA^/- -^SEA^
F28	*HBA*	7.0	51	0	21	0	M_p_	P_p_	100	100	- -^SEA^/αα^WS^	- -^SEA^/αα^WS^
F29	*HBA*	16.6	50	0	13	0	M_p_	P_p_	100	100	- -^SEA^/- -^SEA^	- -^SEA^/- -^SEA^
F30	*HBA*	14.5	0	64	0	34	M_n_	P_n_	100	100	N/N	N/N
F31	*HBA*	10.5	52	0	9	0	M_p_	P_p_	100	100	- -^SEA^/- -^SEA^	- -^SEA^/- -^SEA^
F32	*HBA*	16.1	0	38	39	0	M_n_	P_p_	100	100	N/- -^SEA^	N/- -^SEA^
F33	*HBA*	18.4	33	0	0	62	M_p_	P_n_	100	100	- -^SEA^/N	- -^SEA^/N
F34	*HBA*	16.5	0	20	13	0	M_n_	P_p_	100	100	N/- -^SEA^	N/- -^SEA^
F35	*HBA*	14.3	47	0	0	28	M_p_	P_n_	100	100	- -^SEA^/N	- -^SEA^/N
F36	*HBA*	20.6	49	0	0	17	M_p_	P_n_	100	100	- -^SEA^/N	- -^SEA^/N
F37	*HBA*	20.6	8	0	11	0	M_p_	P_p_	100	100	- -^SEA^/- -^SEA^	- -^SEA^/- -^SEA^
F38	*HBA*	15.3	0	51	0	9	M_n_	P_n_	100	100	N/N	N/N
F39	*HBA*	23.9	0	36	0	25	M_n_	P_n_	100	100	N/N	N/N
F40	*HBA*	18.3	47	0	0	7	M_p_	P_n_	100	100	- -^SEA^/N	- -^SEA^/N
F41	*HBA*	12.3	0	43	0	34	M_n_	P_n_	100	100	N/N	N/N
F42	*HBA*	17.3	0	33	31	0	M_n_	P_p_	100	100	N/- -^SEA^	N/- -^SEA^
F43	*HBA*	8.8	59	0	0	13	M_p_	P_n_	100	100	- -^SEA^/N	- -^SEA^/N
F44	*HBA*	26.1	31	0	42	0	M_p_	P_p_	100	100	- -^SEA^/- -^SEA^	- -^SEA^/- -^SEA^
F45	*HBA*	24.4	0	80	17	0	M_n_	P_p_	100	100	N/- -^SEA^	N/- -^SEA^
F46	*HBA*	22.4	29	0	0	19	M_p_	P_n_	100	100	- -^SEA^/N	- -^SEA^/N
F47	*HBA*	15.5	35	0	0	5	M_p_	P_n_	100	100	- -^SEA^/N	- -^SEA^/N
F48	*HBA*	11.8	0	32	0	54	M_n_	P_n_	100	100	N/N	N/N
F50	*HBA*	16.2	49	0	4	0	M_p_	P_p_	100	100	- -^SEA^/- -^SEA^	- -^SEA^/- -^SEA^
F51	*HBA*	13.7	40	0	0	35	M_p_	P_n_	100	100	- -^SEA^/N	- -^SEA^/N
F52	*HBA*	10.7	0	42	27	0	M_n_	P_p_	100	100	N/αα^CS^	N/αα^CS^
F54	*HBA*	12.8	0	44	42	0	M_n_	P_p_	100	100	N/- -^SEA^	N/- -^SEA^
F56	*HBA*	18.0	0	21	0	8	M_n_	P_n_	100	100	N/N	N/N
**7 fetuses received only one allele genotype**												
F23	*HBB*	14.0	8	0	2	0	M_p_	NC [*]	99	98	c.316-197C>T/NC	c.316-197C>T/c.316-197C>T
F49	*HBA*	13.6	0	1	3	0	NC [*]	P_p_	83	100	NC/- -^SEA^	- -^SEA^/- -^SEA^
F53	*HBA*	21.9	0	1	0	35	NC [*]	P_n_	89	100	NC/N	N/N
F55	*HBA*	9.0	33	0	0	1	M_p_ [**]	NC [*]	100	79	- -^SEA^/NC	N/αα^CS^
F57	*HBA*	21.4	37	0	0	0	M_p_	NC [*]	100	0	- -^SEA^/NC	- -^SEA^/N
F58	*HBA*	22.9	61	0	0	0	M_p_	NC [*]	100	0	- -^SEA^/NC	- -^SEA^/- -^SEA^
F59	*HBA*	14.4	13	0	1	0	M_p_	NC [*]	100	93	- -^SEA^/NC	- -^SEA^/- -^SEA^

*Abbreviations*: *FF*, fetal fraction; *No*., number; *NIPT*, noninvasive prenatal testing; *N*, normal allele; *NC*, no-call; *Hb CS, HBA2* c.427T>C; *Hb Westmead (WS)*, *HBA2*:c.369C>G; *Mat Hap*, fetal inheritance from maternal haplotype; *Pat Hap*, fetal inheritance from paternal haplotype; ${\mathit{\mathsf{CS}}}_{\mathit{\mathsf{mat}}}$, confidence score for fetal inheritance from maternal haplotype; ${\mathit{\mathsf{CS}}}_{\mathit{\mathsf{pat}}}$, confidence score for fetal inheritance from paternal haplotype; *M*_*p*_, maternal pathogenic haplotype, *P*_*p*_, paternal pathogenic haplotype; *M*_*n*_, maternal normal haplotype; *P*_*n*_, paternal normal haplotype; *SNPs for M*_*p*_*/P*_*p*_, the number of informative SNPs that supported fetal inheritance from parental pathogenic haplotypes; *SNPs for M*_*n*_*/P*_*n*_; the number of informative SNPs that supported fetal inheritance from parental normal haplotypes

*No-call: confidence score less than 0.99. **The NIPT result of maternal inheritance for F55 was inconsistent with the invasive prenatal diagnosis result

**Fig. 3:**
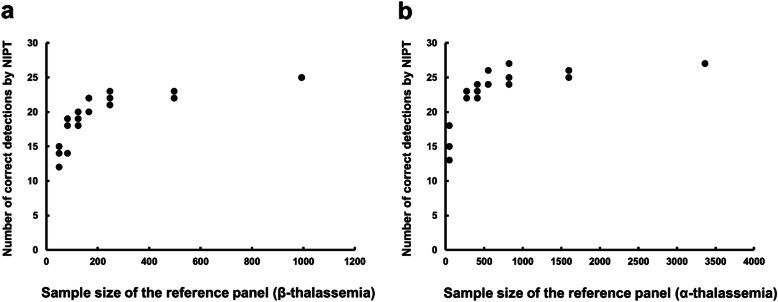
Effect of the reference panel sample size on the outcomes of PBH-NIPT. We randomly extracted one-half, one-quarter, one-sixth, one-eighth, one-twelfth, and 50 of the samples from the total reference panel and performed three independent tests for **a** β-thalassemia and **b** α-thalassemia. In the 52 fetuses in whom NIPT successfully inferred both alleles, the outcome of NIPT improved as the sample size of the reference panel increased

To evaluate the relationship between the accuracy of NIPT and the reference panel sample size, we randomly selected one-half, one-quarter, one-sixth, one-eighth, one-twelfth, and 50 of the samples from the total reference panel and performed three independent tests. As expected, in the 52 fetuses in whom NIPT inferred both alleles, the NIPT outcome improved as the reference panel sample size increased (Fig. 3). Reduction of the sample size to one-half of the total reference panel yielded accuracies of NIPT of approximately 89.3% for β-thalassemia and 95.1% for α-thalassemia relative to the invasive prenatal diagnosis results.

## Discussion

This study demonstrated the feasibility of PBH-NIPT for thalassemia. PBH-NIPT can be used after carrier screening for thalassemia. For high-risk couples reluctant to undergo an invasive procedure, PBH-NIPT is a more attractive option, requiring only a simple blood draw from the pregnant woman. For most conditions, including the deduction of carrier and normal individuals using NIPT, no further confirmation is needed. For conditions where NIPT detects an affected fetus (12 cases) or detects only one pathogenic allele (6 cases), invasive prenatal diagnosis is recommended. In our study, PBH-NIPT dramatically reduced the number of invasive prenatal diagnosis required by approximately 69.5% (from 59 to 18 fetuses).

Here, NIPT successfully inferred 94.1% of the fetal alleles (111/118) from the 59 fetuses. Focusing on the 7 no-call cases clearly shows that the number of informative SNPs in all 7 cases was fewer than 3. This problem can be resolved by increasing the number of informative SNPs flanking the target gene through expansion of the target region [33]. Moreover, this study demonstrated that the reference panel size could affect the performance of NIPT. However, the reference panel size is not a limiting factor since large-scale expanded carrier screening for recessive monogenic disorders is common in clinical practice [40].

This study aimed to evaluate and provide a simple, fast, and inexpensive NIPT method for thalassemia. Compared with the current linked-read sequencing-based NIPT method, which requires 15–20 days (10 days of wet lab work and 5–10 days of data analysis) [25], PBH-NIPT requires only 5–7 days (4–5 days of wet lab work and 1–2 days of data analysis). Training PBH on a large reference panel requires only a few minutes [41]. The PBH-NIPT method costs approximately 80 dollars, as estimated in the supplement (Additional file 8: Table S4). Considering the cost of invasive prenatal diagnosis (~ 1000 dollars/sample [42]) testing in 6 cases, the actual cost of PBH-NIPT per sample is 174 dollars, which is significantly less than those of molecular haplotyping (~ 1500 dollars/sample [25]) and invasive prenatal diagnosis (~ 1000 dollars/sample [42]).

This study has two limitations. First, since all 59 families and training reference data were from southern China, the test cannot detect individuals with ethnic backgrounds differing from those in the training population. Currently, we can only consult ethnic information based on self-reports before testing. A potentially good solution would be to add a quantifiable QC parameter to provide guidance for the reliability of the test. Therefore, we will consider including SNPs that are able to distinguish ethnic information when designing the next version of the probe [43]. Second, the population frequency of these 10 variants was 0.15~2.66% in our dataset [35], and more data are needed to validate whether PBH-NIPT is able to detect variants with lower frequencies.

## Conclusions

In summary, we developed and verified PBH-NIPT, a novel method for prenatal testing of α-thalassemia and β-thalassemia. Compared with invasive prenatal diagnosis, this method achieved 99.1% accuracy (95% CI, 95.1–100%). Therefore, we propose that this strategy might be extended to detect variants in addition to single-haplotype founder variants in other recessive monogenic disorders. Additional studies with larger sample sizes are required to confirm the application and performance of PBH-NIPT for other populations and variants with lower frequencies.

## Supplementary Information

**Additional file 1: Fig. S1.** Variant spectrum of thalassemia in our published data [35].

**Additional file 2: Table S1.** Clinical information of the participating families.

**Additional file 3: Supplementary Methods.**

**Additional file 4: Table S2.** Characteristics of the 59 families.

**Additional file 5: Fig. S2.** Concordance of parental haplotypes deduced by PBH and FBH.

**Additional file 6: Table S3.** Concordance of parental haplotypes deduced by PBH and FBH.

**Additional file 7: Fig. S3.** NIPT results for thalassemia.

**Additional file 8: Table S4.** Cost and turnaround time of PBH-NIPT.

**Additional file 9: Source data for Fig. 3.**

**Additional file 10: Source data for Fig. S1.**

## Data Availability

The datasets supporting the conclusions of this article are included within the article and its additional files. Nonidentifiable data of 59 families generated during this study are deposited in the CNGB Nucleotide Sequence Archive (CNSA: https://db.cngb.org/search/project/CNP0000644/) with accession number CNP0000644 [44]. The above data have also been deposited in the European Variation Archive (EVA: https://wwwdev.ebi.ac.uk/eva/?eva-study=PRJEB42529) with accession number PRJEB42529 [45]. The raw data of 4356 samples, which was not open to the public in the original paper [35], cannot be submitted to public databases because the patients were not consented for sharing their raw data. The source data used to generate Fig. 3 and Fig. S1 are also available. The customized scripts for PBH and NIPT can be found at https://github.com/liserjrqlxue/NIPT-Thalassemia. The following open software were used: Beagle 4.0 (https://faculty.washington.edu/browning/beagle/b4_0.html) [46] BWA 0.7.12 (http://bio-bwa.sourceforge.net/) [47] Picard 1.87 (http://broadinstitute.github.io/picard/) [48]

## Abbreviations

NIPT: Noninvasive prenatal testing
FBH: Family-based haplotyping
PBH-NIPT: Population-based haplotyping-NIPT
HMM: Hidden Markov model
cfDNA: Cell-free DNA
RMD: Relative mutation dosage
RHDO: Relative haplotype dosage
SNVs: Single nucleotide variants
InDels: Small insertions/deletions
CNVs: Copy number variants
TLA: Targeted locus amplification
FF: Fetal fraction
gDNA: Genomic DNA
SNPs: Single-nucleotide polymorphisms
CS: Confidence score
AF: Amniotic fluid
CVS: Chorionic villus sample
PCR: Polymerase chain reaction
ssDNA: Single-stranded DNA
DNBs: DNA nanoballs
RDB-PCR: Reverse dot blot PCR
