# Supplementary material for: Noninvasive prenatal testing of α-thalassemia and β-thalassemia through population-based parental haplotyping
Source: Genome Med. 2021 Feb 5;13:18. doi: 10.1186/s13073-021-00836-8 (PMC7866698; doi:10.1186/s13073-021-00836-8)
Supplement: Supplementary file 1 — Additional file 1: Fig. S1. Variant spectrum of thalassemia in our published data [35]. [file 13073_2021_836_MOESM1_ESM.docx]

**
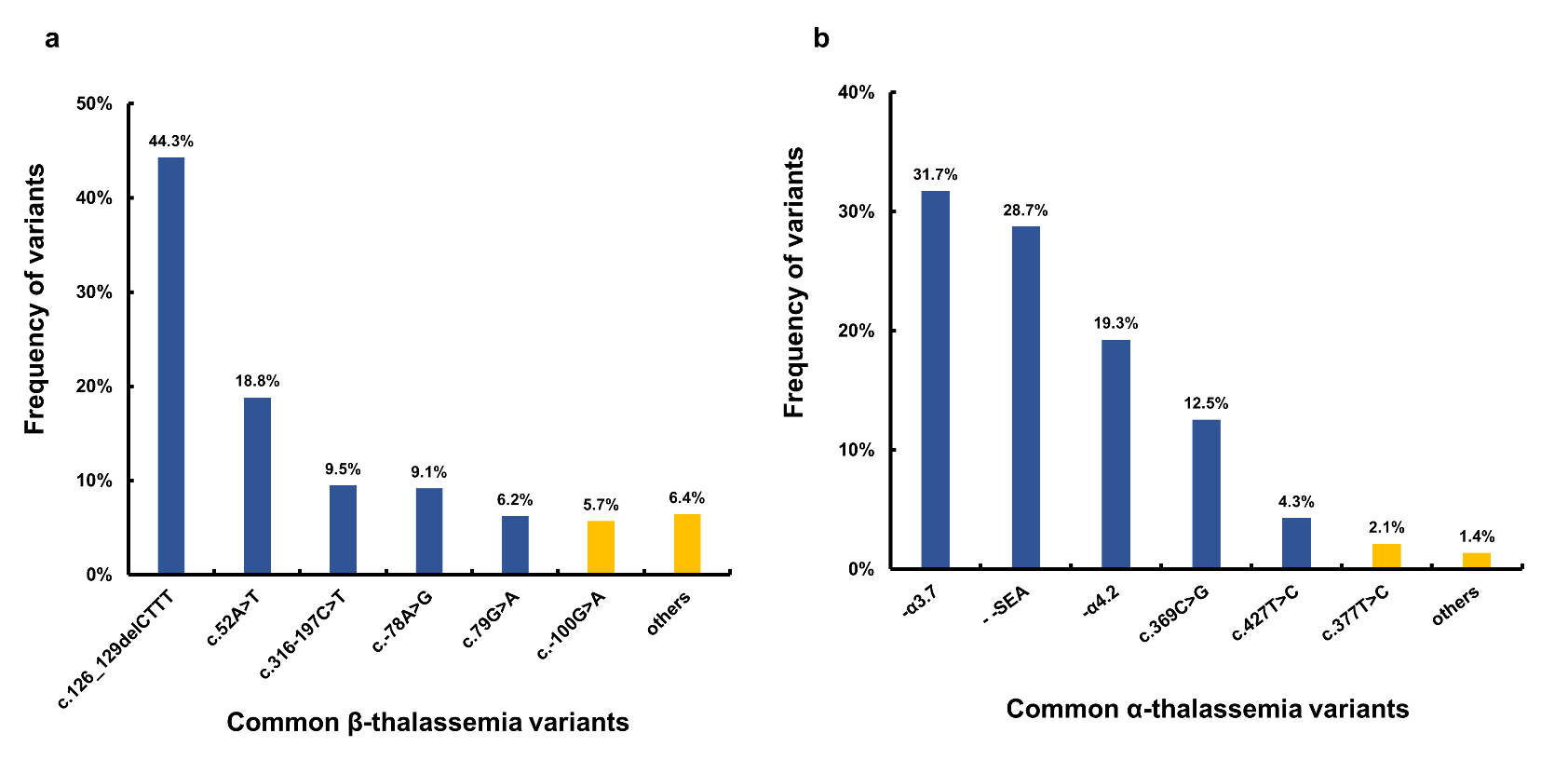
**

**Additional file 1: Figure S1.** **Variant spectrum of thalassemia** **in our published data [**[**35**](#_ENREF_35)**].**

**a,** Variant spectrum of β-thalassemia. The blue bar indicates the top five variants in the *HBB* (NM_000518.4) gene, which account for 87.9% of β-thalassemia carriers. **b,** Variant spectrum of α-thalassemia. The blue bar indicates the top five variants in the *HBA1* (NM_000558.3) and *HBA2* (NM_000517.4) genes, which account for 96.5% of α-thalassemia carriers.
