# Supplementary material for: Noninvasive prenatal testing of α-thalassemia and β-thalassemia through population-based parental haplotyping
Source: Genome Med. 2021 Feb 5;13:18. doi: 10.1186/s13073-021-00836-8 (PMC7866698; doi:10.1186/s13073-021-00836-8)
Supplement: Supplementary file 2 — Additional file 2: Table S1. Clinical information of the participating families. [file 13073_2021_836_MOESM2_ESM.docx]

**Additional file 2: Table S1 Clinical information of** **the participating families**

| **Family** | **Disease** | **Gene** | **Genotypes of the Trios** | | | **GW** |
| --- | --- | --- | --- | --- | --- | --- |
|  |  |  | **Mat** | **Pat** | **Fetus (Mat/Pat)** |  |
| F01 | β-thalassemia | *HBB* | c.52A>T/N | c.126_129delCTTT/N | c.52A>T/c.126_129delCTTT | 22 |
| F02 | β-thalassemia | *HBB* | c.316-197C>T/N | c.-78A>G/N | N/N | 12^+4^ |
| F03 | β-thalassemia | *HBB* | c.126_129delCTTT/N | c.126_129delCTTT/N | N/N | 20^+5^ |
| F04 | β-thalassemia | *HBB* | c.126_129delCTTT/N | c.126_129delCTTT/N | c.126_129delCTTT/c.126_129delCTTT | 16 |
| F05 | β-thalassemia | *HBB* | c.126_129delCTTT/N | c.126_129delCTTT/N | N/c.126_129delCTTT | 17 |
| F06 | β-thalassemia | *HBB* | c.126_129delCTTT/N | c.126_129delCTTT/N | c.126_129delCTTT/N | 12^+1^ |
| F07 | β-thalassemia | *HBB* | c.316-197C>T/N | c.126_129delCTTT/N | N/c.126_129delCTTT | 18 |
| F08 | β-thalassemia | *HBB* | c.126_129delCTTT/N | c.316-197C>T/N | c.126_129delCTTT/N | 13^+6^ |
| F09 | β-thalassemia | *HBB* | c.126_129delCTTT/N | c.126_129delCTTT/N | c.126_129delCTTT/N | 12^+6^ |
| F10 | β-thalassemia | *HBB* | c.126_129delCTTT/N | c.316-197C>T/N | c.126_129delCTTT/N | 12^+3^ |
| F11 | β-thalassemia | *HBB* | c.52A>T/N | c.126_129delCTTT/N | N/N | 13 |
| F12 | β-thalassemia | *HBB* | c.52A>T/N | c.126_129delCTTT/N | N/N | 13 |
| F13 | β-thalassemia | *HBB* | c.79G>A/N | c.126_129delCTTT/N | N/c.126_129delCTTT | 11^+3^ |
| F14 | β-thalassemia | *HBB* | c.126_129delCTTT/c.-78A>G | c.-78A>G/N | c.126_129delCTTT/c.-78A>G | 12^+1^ |
| F15 | β-thalassemia | *HBB* | c.316-197C>T/N | c.126_129delCTTT/N | c.316-197C>T/N | 12^+1^ |

**Additional file 2: Table S1 Clinical information of the participating families (continued)**

| **Family** | **Disease** | **Gene** | **Genotypes of the Trios** | | | **GW** |
| --- | --- | --- | --- | --- | --- | --- |
|  |  |  | **Mat** | **Pat** | **Fetus (Mat/Pat)** |  |
| F16 | β-thalassemia | *HBB* | c.316-197C>T/N | c.316-197C>T/N | c.316-197C>T/N | 12^+2^ |
| F17 | β-thalassemia | *HBB* | c.316-197C>T/N | c.-78A>G/N | c.316-197C>T/N | 11^+5^ |
| F18 | β-thalassemia | *HBB* | c.-78A>G/N | c.52A>T/N | c.-78A>G/N | 12^+5^ |
| F19 | β-thalassemia | *HBB* | c.126_129delCTTT/N | c.316-197C>T/N | N/N | 13^+5^ |
| F20 | β-thalassemia | *HBB* | c.316-197C>T/N | c.316-197C>T/N | N/N | 14^+2^ |
| F21 | β-thalassemia | *HBB* | c.52A>T/N | c.126_129delCTTT/N | N/N | 12 |
| F22 | β-thalassemia | *HBB* | c.52A>T/N | c.126_129delCTTT/N | c.52A>T/c.126_129delCTTT | 11^+6^ |
| F23 | β-thalassemia | *HBB* | c.316-197C>T/N | c.316-197C>T/N | c.316-197C>T/c.316-197C>T | 12^+1^ |
| F24 | β-thalassemia | *HBB* | c.126_129delCTTT/N | c.-78A>G/N | N/N | 12^+5^ |
| F25 | β-thalassemia | *HBB* | c.126_129delCTTT/N | c.316-197C>T/N | N/N | 11^+6^ |
| F26 | β-thalassemia | *HBB* | c.126_129delCTTT/N | c.-78A>G/N | c.126_129delCTTT/c.-78A>G | 12^+3^ |
| F27 | α-thalassemia | *HBA* | - -^SEA^/N | - -^SEA^/N | - -^SEA^/- -^SEA^ | 21 |
| F28 | α-thalassemia | *HBA* | - -^SEA^/N | αα^WS^/N | - -^SEA^/αα^WS^ | 18 |
| F29 | α-thalassemia | *HBA* | - -^SEA^/N | - -^SEA^/N | - -^SEA^/- -^SEA^ | 13^+3^ |
| F30 | α-thalassemia | *HBA* | - -^SEA^/N | - -^SEA^/N | N/N | 11^+6^ |

**Additional file 2: Table S1 Clinical information of the participating families (continued)**

| **Family** | **Disease** | **Gene** | **Genotypes of the Trios** | | | **GW** |
| --- | --- | --- | --- | --- | --- | --- |
|  |  |  | **Mat** | **Pat** | **Fetus (Mat/Pat)** |  |
| F31 | α-thalassemia | *HBA* | - -^SEA^/-α^3.7^ | - -^SEA^/N | - -^SEA^/- -^SEA^ | 11^+6^ |
| F32 | α-thalassemia | *HBA* | - -^SEA^/N | - -^SEA^/N | N/- -^SEA^ | 12 |
| F33 | α-thalassemia | *HBA* | - -^SEA^/N | - -^SEA^/N | - -^SEA^/N | 13^+1^ |
| F34 | α-thalassemia | *HBA* | - -^SEA^/N | - -^SEA^/N | N/- -^SEA^ | 12^+5^ |
| F35 | α-thalassemia | *HBA* | - -^SEA^/N | - -^SEA^/N | - -^SEA^/N | 10^+6^ |
| F36 | α-thalassemia | *HBA* | - -^SEA^/N | - -^SEA^/N | - -^SEA^/N | 11^+6^ |
| F37 | α-thalassemia | *HBA* | - -^SEA^/N | - -^SEA^/N | - -^SEA^/- -^SEA^ | 12^+2^ |
| F38 | α-thalassemia | *HBA* | - -^SEA^/N | - -^SEA^/N | N/N | 11^+5^ |
| F39 | α-thalassemia | *HBA* | - -^SEA^/N | - -^SEA^/N | N/N | 10^+3^ |
| F40 | α-thalassemia | *HBA* | - -^SEA^/N | - -^SEA^/N | - -^SEA^/N | 15^+5^ |
| F41 | α-thalassemia | *HBA* | - -^SEA^/N | αα^CS^/N | N/N | 13^+4^ |
| F42 | α-thalassemia | *HBA* | - -^SEA^/N | - -^SEA^/N | N/- -^SEA^ | 11^+4^ |
| F43 | α-thalassemia | *HBA* | - -^SEA^/N | - -^SEA^/N | - -^SEA^/N | 11^+2^ |
| F44 | α-thalassemia | *HBA* | - -^SEA^/N | - -^SEA^/N | - -^SEA^/- -^SEA^ | 11^+3^ |
| F45 | α-thalassemia | *HBA* | - -^SEA^/N | - -^SEA^/N | N/- -^SEA^ | 10^+6^ |
| F46 | α-thalassemia | *HBA* | - -^SEA^/N | -α^3.7^/N | - -^SEA^/N | 11^+6^ |
| F47 | α-thalassemia | *HBA* | - -^SEA^/N | - -^SEA^/N | - -^SEA^/N | 13 |

**Additional file 2: Table S1 Clinical information of the participating families (continued)**

| **Family** | **Disease** | **Gene** | **Genotypes of the Trios** | | | **GW** |
| --- | --- | --- | --- | --- | --- | --- |
|  |  |  | **Mat** | **Pat** | **Fetus (Mat/Pat)** |  |
| F48 | α-thalassemia | *HBA* | - -^SEA^/N | - -^SEA^/N | N/N | 12 |
| F49 | α-thalassemia | *HBA* | - -^SEA^/N | - -^SEA^/N | - -^SEA^/- -^SEA^ | 12 |
| F50 | α-thalassemia | *HBA* | - -^SEA^/N | - -^SEA^/-α^3.7^ | - -^SEA^/- -^SEA^ | 13^+5^ |
| F51 | α-thalassemia | *HBA* | - -^SEA^/N | - -^SEA^/N | - -^SEA^/N | 12^+6^ |
| F52 | α-thalassemia | *HBA* | -α^3.7^/N | - -^SEA^/αα^CS^ | N/αα^CS^ | 11^+1^ |
| F53 | α-thalassemia | *HBA* | - -^SEA^/N | - -^SEA^/N | N/N | 10^+3^ |
| F54 | α-thalassemia | *HBA* | -α^4.2^/N | - -^SEA^/N | N/- -^SEA^ | 12^+5^ |
| F55 | α-thalassemia | *HBA* | - -^SEA^/N | αα^CS^/N | N/αα^CS^ | 12^+2^ |
| F56 | α-thalassemia | *HBA* | - -^SEA^/N | - -^SEA^/N | N/N | 13^+2^ |
| F57 | α-thalassemia | *HBA* | - -^SEA^/N | - -^SEA^/N | - -^SEA^/N | 10^+6^ |
| F58 | α-thalassemia | *HBA* | - -^SEA^/N | - -^SEA^/N | - -^SEA^/- -^SEA^ | 13^+5^ |
| F59 | α-thalassemia | *HBA* | - -^SEA^/N | - -^SEA^/N | - -^SEA^/- -^SEA^ | 10^+1^ |

Abbreviations: N, normal allele; GW, gestational weeks; CS, *HBA2* c.427T>C; WS, *HBA2* c.369C>G.
