# Supplementary material for: Noninvasive prenatal testing of α-thalassemia and β-thalassemia through population-based parental haplotyping
Source: Genome Med. 2021 Feb 5;13:18. doi: 10.1186/s13073-021-00836-8 (PMC7866698; doi:10.1186/s13073-021-00836-8)
Supplement: Supplementary file 3 — Additional file 3: Supplementary Methods. [file 13073_2021_836_MOESM3_ESM.docx]

**Noninvasive prenatal** **testing of** **α-thalassemia and β-thalassemia through population-based parental haplotyping**

Chao Chen^1,2,6^, Ru Li^3,6^, Jun Sun^1,2,6^, Yaping Zhu^1,2^, Lu Jiang^1,2^, Jian Li^3^, Fang Fu^3^, Junhui Wan^3^, Fengyu Guo^1,2^, Xiaoying An^1,2^, Yaoshen Wang^1,2^, Linlin Fan^1,2^, Yan Sun^1,4^, Xiaosen Guo^1^, Sumin Zhao^1,2^, Wanyang Wang^1,2^, Fanwei Zeng^1^, Yun Yang^1,4,5^, Peixiang Ni^1,2^, Yi Ding^1,2^, Bixia Xiang^1^, Zhiyu Peng^1*^, and Can Liao^3*^

^1^BGI Genomics, BGI-Shenzhen, Shenzhen 518083, China;

^2^Tianjin Medical Laboratory, BGI-Tianjin, BGI-Shenzhen, Tianjin 300308, China;

^3^Department of Prenatal Diagnostic Center, Guangzhou Women and Children's Medical Center, Guangzhou Medical University, Guangzhou 510623, China;

^4^BGI-Wuhan Clinical Laboratories, BGI-Shenzhen, Wuhan 490079, China;

^5^Department of Obstetrics and Gynecology, The Second Affiliated Hospital of Zhengzhou University, Zhengzhou 450052, China.

^6^These authors contributed equally to this work.

*Correspondence: canliao6008@163.com or pengzhiyu@bgi.com

**Supplementary Methods**

**Classification of parental SNPs**

Parental SNPs with a sequencing depth of at least 30X were extracted and classified into 4 groups based on the following 4 possible types of parental allele combinations (Table S1).

**Table S1. Four groups of parental SNP combinations.**

| **Group** | **Paternal genotype** | **Maternal genotype** |
| --- | --- | --- |
| Type 1 (for fetal fraction estimation) | A/A | B/B |
|  | B/B | A/A |
| Type 2 (Paternal-specific) | A/B | A/A |
|  | A/B | B/B |
| Type 3 (Maternal-specific) | A/A | A/B |
|  | B/B | A/B |
| Type 4 | A/B | A/B |

For illustration, the biallelic sites are shown as carrying the “A” or “B” allele.

**Fetal fraction (FF) estimation**

We used type 1 SNPs (Table S1) with a sequencing depth of greater than 30X in maternal plasma to calculate the FF. The FF was estimated using the following equation:$FF=2\times\frac{\sum d_{f}}{\sum(d_{f}+d_{m})}$, where $d_{f}$ is the number of reads supporting the paternal genotype, and $d_{m}$ is the number of reads supporting the maternal genotype.

**Concordance of haplotypes constructed by population-based haplotyping (PBH) and family-based haplotyping (FBH)**


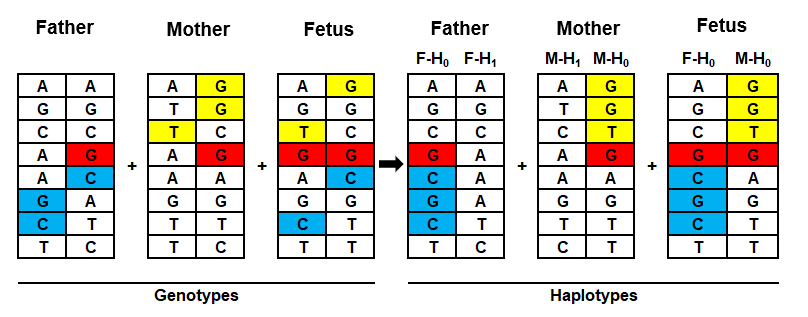
First, we constructed the haplotypes of 59 families using an FBH strategy based on Mendel’s law**^1^** (Figure S1). We inferred the paternal haplotype with type 2 SNPs (Table S1), which were heterozygous in the father but homozygous in the mother, and the maternal haplotype with type 3 SNPs (Table S1), which were heterozygous in the mother but homozygous in the father. We inferred the fetal haplotype according to family-based linkage. We used the genotypes of the father, mother and fetus to generate haplotypes for all families. Note that for F04, we used DNA from the sister instead of the fetus to generate the parental haplotypes because of insufficient fetal DNA.

**Figure S1 Strategy of family-based haplotyping (FBH).** Blue highlight: type 2 SNPs; yellow highlight: type 3 SNPs; red highlight: pathogenic variants; H_0_: haplotype linked to the pathogenic variant; H_1:_ haplotype linked to the normal variant.

Second, we compared the parental haplotypes generated by PBH against those generated by FBH. We calculated the concordance rate as the number of consistent phased SNPs in the haplotypes divided by the number of all phased SNPs in the haplotypes detected by both methods. In addition, we compared the fetal haplotypes inferred by PBH-NIPT against those generated by FBH.

**PBH-NIPT of the fetal genotype at pathogenic sites**

***1. Inferring fetal haplotypes via the hidden Markov model (HMM) and Viterbi algorithm***

We slightly modified a previously published algorithm**^1^** based on an FBH strategy to be applicable in this study (Figure S2). While we obtained the parental haplotypes using population-based haplotyping, we determined the fetal inheritance from the father and mother separately, as follows.

First, we determined paternal inheritance using paternal informative SNPs (type 2 SNPs, Table S1), which are heterozygous in the father but homozygous in the mother.

Second, we determined maternal inheritance using maternal informative SNPs, which include two types of SNPs: 1) SNPs heterozygous in the mother but homozygous in the father (type 3 SNPs, Table S1) and 2) SNPs heterozygous in both parents (type 4 SNPs, Table S1) in the blocks where the first step inferred the fetal inherited haplotype from the father. Because the fetal inheritance from the father was already determined in the first step, we could regard type 4 SNPs as type 3 SNPs. For example, if a paternal genotype is A/B and the fetal inheritance from this father is A, then the paternal genotype A/B is equivalent to A/A (paternal) and A/B (maternal). We integrated these two types of SNPs and performed the analysis with the same procedure used for type 3 SNPs.


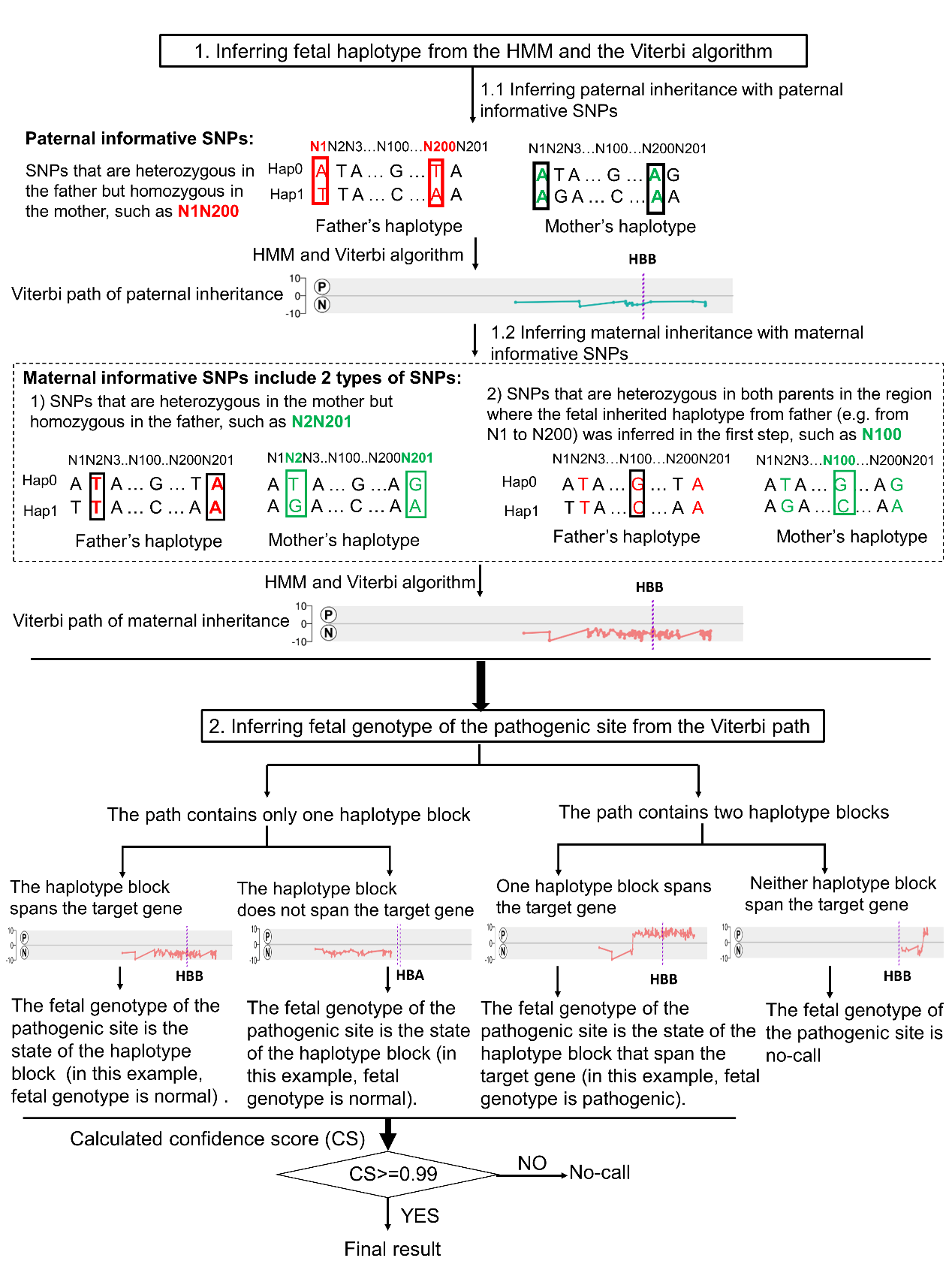
 **Figure S2 Diagram illustrating the principles of PBH-NIPT for thalassemia**

We constructed a hidden Markov model (HMM) and used the Viterbi algorithm (Figure S3) to determine the fetal haplotypes of parental transmitted variants.

**
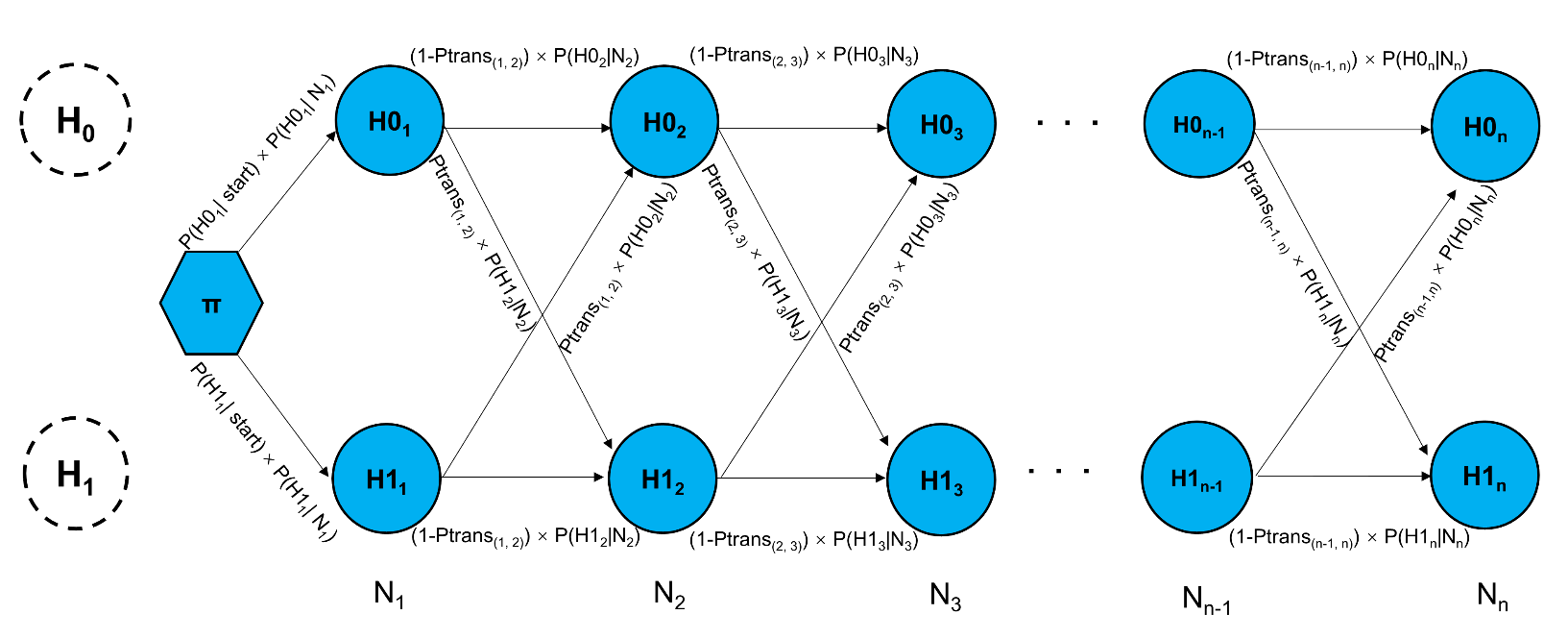
Figure S3 Diagram illustrating the application of the HMM and Viterbi algorithm for NIPT.**

Sites that can be used to infer the fetal haplotype in a certain block were denoted as N, while the total number of sites was denoted as n. For each site, we represented the observed state as S=${\{N}_{j}\}$ (Figure S3), where $N_{j}$=$\text{\{Pos, }\text{Fht}\text{, }\text{Mht}\text{, }\text{dep\_ref}\text{, }\text{dep\_alt}\text{\}}$ and j=1, 2, 3, …, n. Pos, Fht, Mht, dep_ref, and dep_alt indicate the genomic position, paternal haplotype, maternal haplotype, and the number of maternal plasma sequencing reads supporting the reference and nonreference alleles, respectively.

We defined H_0_ as the pathogenic haplotype, which is linked to the known pathogenic variant, and H_1_ as the normal haplotype, which is linked to the normal allele. We defined the hidden state by whether the pathogenic haplotype or normal haplotype was transmitted to the fetus and was denoted as $Q=\{H_{0},H_{1} \}$ (Figure S3). The initial state distribution was defined as $\pi=\{P\left( H_{0} \right),P\left( H_{1} \right)\}=\{0.5, 0.5\}$, as the lack of prior probability.

We represented the emission probabilities matrix using the binomial distribution $B=\{b_{i,j}\}$, where $b_{i,j}=P\{H_{i}|N_{j}\}$; i=0,1; and j=1, 2, 3, …, n. We calculated the probabilities that $H_{i}$ was transmitted to the fetus for the given sites j using the following Bayes formula:

$$P_{\mathrm{emission}}=P\{H_{i}|N_{j}\}=\frac{P\{N_{j}|H_{i}\}\times0.5}{P\{N_{j}|H_{0}\}\times0.5+P\{N_{j}|H_{1}\}\times0.5}=\frac{P\{N_{j}|H_{i}\}}{P\{N_{j}|H_{0}\}+P\{N_{j}|H_{1}\}}.$$

We calculated P{$N_{j}$|$H_{0}$} and P{$N_{j}$|$H_{1}$} using a binomial distribution:

$$P\{N_{j}|H_{0}\}=b(k,n,\mathrm{Prob}_{H0})$$

$$P\{N_{j}|H_{1}\}=b(k,n,\mathrm{Prob}_{H1})$$

where P {$N_{j}$|$H_{i}$} is the probability of the observed state $N_{j}$ at a given SNP site j in the plasma when $H_{i}$ was transmitted to the fetus, k is the observed reads of the target haplotype allele, n is the total number of reads at a given site, and $\mathrm{Prob}_{H0}$ and $\mathrm{Prob}_{H1}$ are the expected probabilities of the target haplotype alleles calculated according to the FF (ϵ).

For predictions of paternal inheritance, we predicted inheritance of paternal informative SNPs based on a binomial distribution. If the paternal-specific allele is transmitted, the expected probability is ϵ/2. Similarly, if the paternal-specific allele is not transmitted, the expected probability is 0. However, due to sequencing and mapping errors, we allowed a low probability (<0.02) of observing the paternal-specific allele even without transmission. The expected probability ($\mathrm{Prob}_{\mathrm{Hi}}$) for each site under different conditions was calculated as follows. We defined the reference allele as 0 and the alternative allele as 1. We denoted the read number supporting the reference allele in maternal plasma as Mp_ref and the read number supporting the alternative allele in maternal plasma as Mp_alt.

| **Paternal**  **H_0_ allele** | **Paternal**  **H_1_ allele** | **Maternal inherited allele** | **No. of reads supporting the paternal-specific allele (k)** | $\mathbf{Prob}_{\mathbf{H0}}$ | $\mathbf{Prob}_{\mathbf{H1}}$ |
| --- | --- | --- | --- | --- | --- |
| 0 | 1 | 0 | Mp_alt | 0.02 | ϵ/2 |
| 1 | 0 | 0 | Mp_alt | ϵ/2 | 0.02 |
| 0 | 1 | 1 | Mp_ref | ϵ/2 | 0.02 |
| 1 | 0 | 1 | Mp_ref | 0.02 | ϵ/2 |

For predictions of maternal inheritance, we similarly predicted inheritance of maternal informative SNPs with a binomial model. We used all heterozygous SNPs detected in the mother’s white blood cells to calculate the ratio of alternative alleles and calculated the mean value as HetAltRatio with the following equation: HetAltRatio=$\frac{\sum_{j=1}^{n} \frac{{RC alt}_{j}}{\mathrm{RC}_{j}}}{n}$, where $\mathrm{RC}_{j}$ represents the read counts at the specific site j and RC alt_j_ represents the read counts of the alternative allele. If the maternally inherited allele is identical to the paternal allele at a given maternal informative site, the expected probability is HetAltRatio*(1-ϵ) for the inherited reference allele or HetAltRatio*(1-ϵ)+ϵ for the inherited alternative allele. If the maternally inherited allele and the paternal allele differ at a given maternal informative site, the expected probability is HetAltRatio. The details of the expected probability ($\mathrm{Prob}_{\mathrm{Hi}}$) for each site under different conditions are as follows. We denoted the read number supporting the alternative allele in maternal plasma as Mp_alt.

| **Maternal**  **H_0_ allele** | **Maternal**  **H_1_ allele** | **Paternal  inherited allele** | **No. of reads supporting the alternative allele (k)** | $\mathbf{Prob}_{\mathbf{H0}}$ | $\mathbf{Prob}_{\mathbf{H1}}$ |
| --- | --- | --- | --- | --- | --- |
| 0 | 1 | 0 | Mp_alt | HetAltRatio*(1-ϵ) | HetAltRatio |
| 1 | 0 | 0 | Mp_alt | HetAltRatio | HetAltRatio*(1-ϵ) |
| 0 | 1 | 1 | Mp_alt | HetAltRatio | HetAltRatio*(1-ϵ)+ϵ |
| 1 | 0 | 1 | Mp_alt | HetAltRatio*(1-ϵ)+ϵ | HetAltRatio |

The transition probability matrix was defined as

$$A=\left\{ a_{i,j;i^{'}j^{'}} \right\},a_{i,j;i^{'},j^{'}}=\left\{ \begin{aligned} P_{\mathrm{trans}\left( j,j^{'} \right)}, &i\neq i^{'},j^{'}=j+1 \\ 1-P_{\mathrm{trans}\left( j,j^{'} \right)}, &i=i^{'},j^{'}=j+1 \\ 0, &j^{'}\neq j+1 \end{aligned} \right.,$$

$$i,i^{'}\in\left\{ 0,1 \right\},j,j^{'}\in\left\{ 1,2,3,\ldots,n \right\}.$$

$P_{trans(j,j')}=\left( \mathrm{cM}_{j^{'}}-\mathrm{cM}_{j} \right)\times1\%$, $P_{trans(j,j')}$ is the probability of recombination between two neighboring SNPs, as calculated by the genetic distance obtained from the HapMap (ftp://ftp.ncbi.nlm.nih.gov/hapmap/recombination/2011-

01_phaseII_B37/genetic_map_HapMapII_GRCh37.tar.gz).

Finally, we used the Viterbi algorithm to determine the most likely path through the observed data and to deduce the inheritance of the parental haplotype using the equation$path=\{r_{1},r_{2},r_{3}...,r_{n}\},r_{i}\in\{0,1\},i\in\{1,2,3...,n\}$, which represents the most likely path (Viterbi path). We determined the Viterbi path was determined as follows.

$$\mathrm{Pat}h=arg max b_{path[n],n}\prod_{j=1}^{n-1} (b_{path[j],j}\times a_{path[j],path[j+1],j}).$$

Extended Data Figure 3 shows the Viterbi paths for each fetus. For each informative SNP j, we could determine the emission probabilities${P_{\mathrm{emission}}}_{0}=P\left\{ H_{0} | N_{j} \right\}$ and ${P_{\mathrm{emission}}}_{1}=P\left\{ H_{1} | N_{j} \right\}$ using the HMM model. The transition probabilities from the previous state (J-1) to the current state J were denoted as${P_{\mathrm{trans}}},$ where

$${P_{\mathrm{trans}}}_{0}=a_{\mathrm{state}\left( j-1 \right),j-1; 0, j}$$

$${P_{\mathrm{trans}}}_{1}=a_{\mathrm{state}\left( j-1 \right),j-1; 1, j}$$

The odds ratio (OR) for the informative SNP j was calculated using the following formula: OR = $ln(\frac{{P_{\mathrm{trans}}}_{0}\times{P_{\mathrm{emission}}}_{0}}{{P_{\mathrm{trans}}}_{1}\times{P_{\mathrm{emission}}}_{1}})$. Adjacent sites were connected with lines.

***2. Inferring fetal genotypes of the pathogenic sites from the Viterbi path***

After obtaining the Viterbi path of fetal haplotypes, we used the following rules to determine the fetal genotypes at pathogenic sites (see Figure S2).

1. If the path contains only one haplotype block (pathogenic OR normal), and
   1.
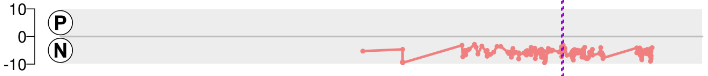
If the haplotype block spans the target gene, the fetal genotype at the pathogenic site is the state of the haplotype block (in this example, the fetal genotype is normal).
   2.
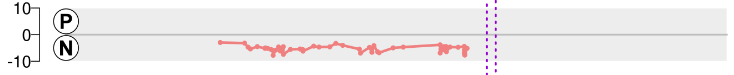
If the haplotype block does not span the target gene due to a lack of informative SNP coverage, we can still safely assume that the phased haplotype block is linked to the adjacent pathogenic site; therefore, the fetal genotype at the pathogenic site is the state of the haplotype block (in this example, the fetal genotype is normal).
2. If the path contains two haplotype blocks (pathogenic AND normal), and
   1.
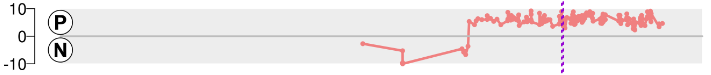
If only one haplotype block spans the target gene, the fetal genotype at the pathogenic site is the state of the haplotype block that spans the target gene (in this example, the fetal genotype is pathogenic).
   2.
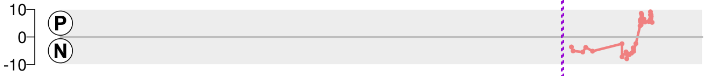
If neither haplotype block spans the target gene, the fetal genotype at the pathogenic site is determined as no-call. In this case, although we can still assume that the pathogenic site is linked to the adjacent site, the haplotype of the adjacent sites is difficult to determine correctly due to possible switch errors (in this example, the fetal genotype is no-call).

Last, we calculated a confidence score (CS) for the fetal genotypes at pathogenic sites inferred by NIPT (via the method described in the next section). Results with confidence scores of less than 0.99 were considered no-call.

***Calculating the confidence score for fetal genotypes at pathogenic sites inferred by NIPT***

To evaluate the probability of obtaining the correct NIPT results for fetal genotypes at pathogenic sites, we used the maternal plasma sequencing depth, FF and number of parental informative SNPs supporting the inferred states (e.g., 43 maternal SNPs for mF08) as the inputs for computational simulation. For each simulation, we first randomly generated a state to represent the correct fetal haplotype. Then, we generated the number of reads in maternal plasma supporting $H_{0}$ and $H_{1}$ in each informative SNP with a binomial distribution. Next, we used a simplified HMM (no recombination events between adjacent sites) and the Viterbi algorithm to obtain the probability that supports the correct fetal haplotype ($P_{\mathrm{true}}$) and the probability that supports the incorrect fetal haplotype ($P_{\mathrm{false}}$). If $P_{\mathrm{true}}>P_{\mathrm{false}}$, we obtained the correct paternal inheritance from NIPT. We repeated this process 100,000 times in each family and denoted the number of correct predictions as N. Thus, the confidence score, which was defined as the probability of obtaining the correct NIPT result, was calculated using the following formula: $\mathrm{CS}=N/{100,000}$.

**References**

1. Ye, J., et al. Haplotype-based Noninvasive Prenatal Diagnosis of Hyperphenylalaninemia through Targeted Sequencing of Maternal Plasma. *Sci Rep* **8,** 161 (2018).
