# Supplementary material for: Noninvasive prenatal testing of α-thalassemia and β-thalassemia through population-based parental haplotyping
Source: Genome Med. 2021 Feb 5;13:18. doi: 10.1186/s13073-021-00836-8 (PMC7866698; doi:10.1186/s13073-021-00836-8)
Supplement: Supplementary file 4 — Additional file 4: Table S2. Characteristics of the 59 families. [file 13073_2021_836_MOESM4_ESM.docx]

**Additional file 4: Table S2. Characteristics of the 59 families.**

| **Variable** | **Value** |
| --- | --- |
| **No. of cases analyzed** | **59** |
| β-thalassemia | 26 |
| α-thalassemia | 33 |
| **Variant allele (β-thalassemia) – no. (%)** | **53** |
| c.126_129delCTTT | 25 (47.2%) |
| c.316-197C>T | 14 (26.4%) |
| c.-78A>G | 7 (13.2%) |
| c.52A>T | 6 (11.3%) |
| c.79G>A | 1 (1.9%) |
| **Variant allele (α-thalassemia) – no. (%)** | **69** |
| - -^SEA^ | 60 (87.0%) |
| -α^3.7^ | 4 (5.8%) |
| CS (*HBA2*: c.427T>C) | 3 (4.3%) |
| -α^4.2^ | 1 (1.4%) |
| WS *(HBA2*: c.369C>G) | 1 (1.4%) |
| **Mean gestational weeks** | 12.6^+3^ (10^+1^ to 22) |
| **Average fetal fraction** | 15.4% (6.0% to 26.1%) |
| **Singleton pregnancy** | 59 |
| **Maternal age (years) – no. (%)** |  |
| 20-25 years | 7 (11.9%) |
| 26-34 years | 45 (76.2%) |
| ≥35 years | 7 (11.9%) |
| **Paternal age (years) – no. (%)** |  |
| 20-25 years | 3 (5.1%) |
| 26-39 years | 52 (88.1%) |
| ≥40 years | 4 (6.8%) |
