# Supplementary material for: Noninvasive prenatal testing of α-thalassemia and β-thalassemia through population-based parental haplotyping
Source: Genome Med. 2021 Feb 5;13:18. doi: 10.1186/s13073-021-00836-8 (PMC7866698; doi:10.1186/s13073-021-00836-8)
Supplement: Supplementary file 5 — Additional file 5: Fig. S2. Concordance of parental haplotypes deduced by PBH and FBH. [file 13073_2021_836_MOESM5_ESM.docx]

**
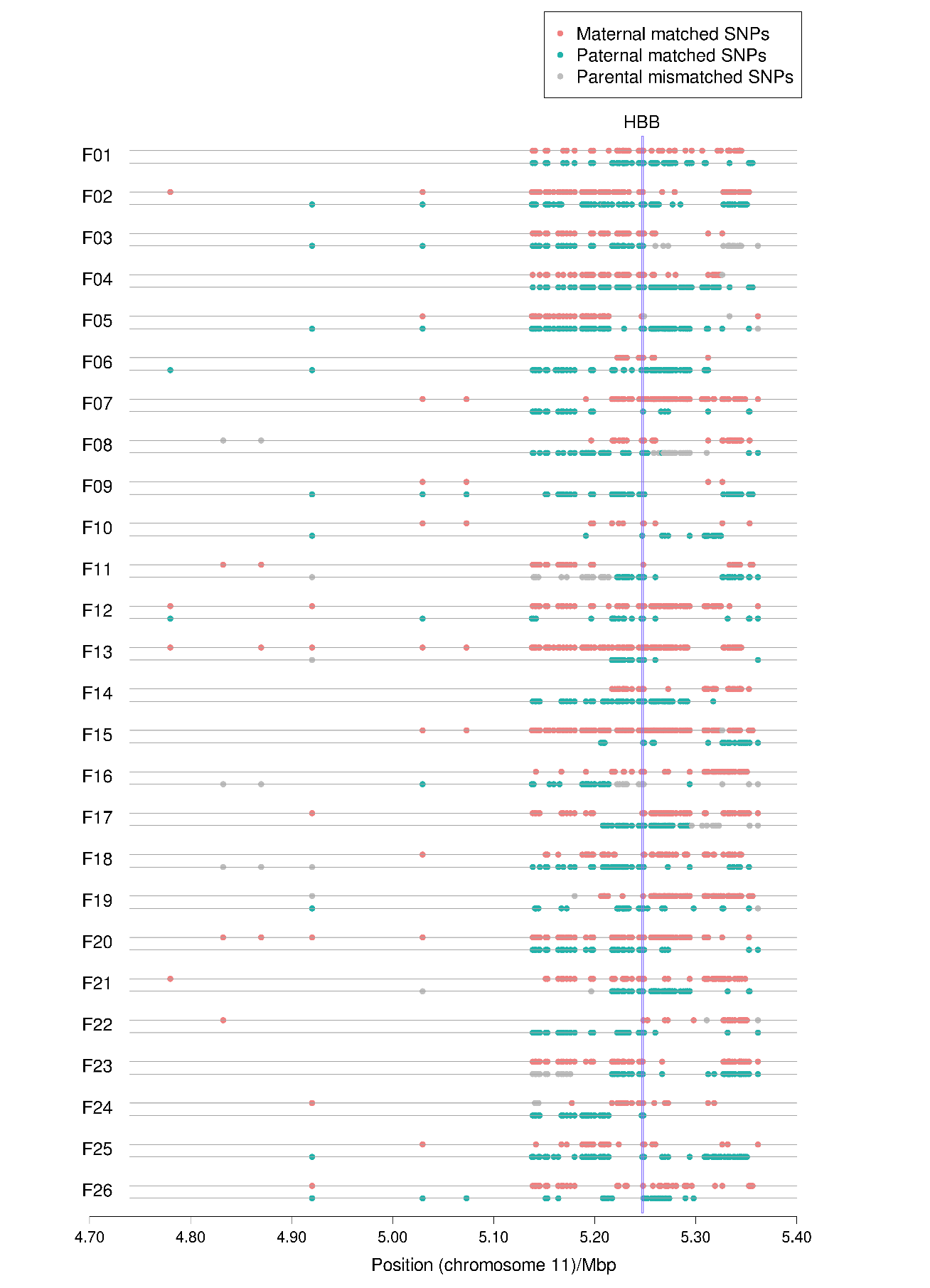
**

**Figure S2a**

**
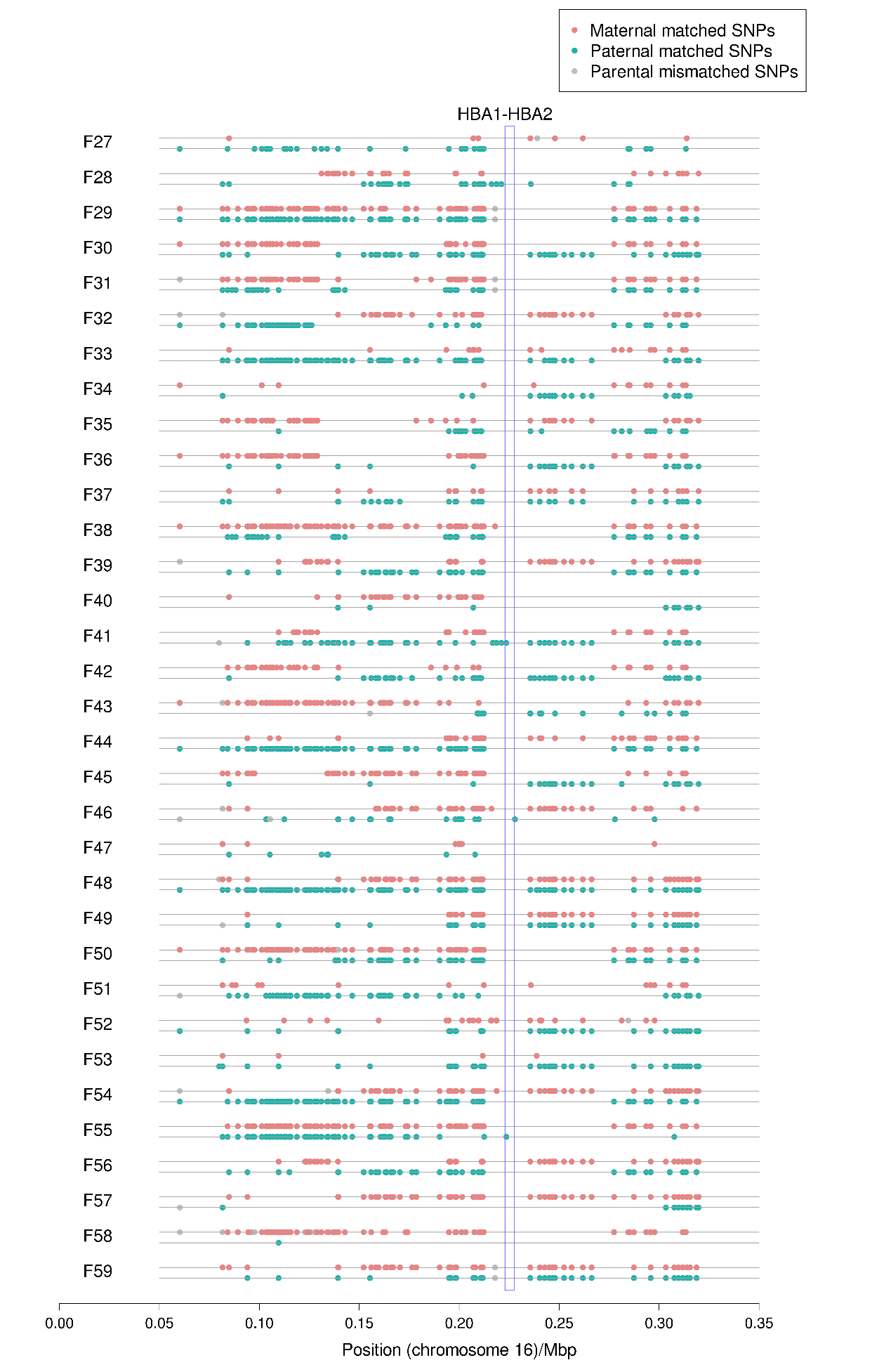
**

**Figure S2b**

**Additional file 5: Figure S2.** **Concordance** **of parental haplotypes deduced by PBH and FBH.**

We compared the parental haplotypes deduced by population-based haplotyping (PBH) and family-based haplotyping (FBH). The red dots and green dots represent the concordant maternal SNPs and paternal SNPs, respectively, phased by these two methods. The gray dots represent inconsistent SNPs phased by these two methods. The x-axis indicates the genomic coordinates of the target region (Mbp). **a,** The concordance of parental haplotypes for β-thalassemia. **b,** The concordance of parental haplotypes for α-thalassemia.
