# Supplementary material for: Noninvasive prenatal testing of α-thalassemia and β-thalassemia through population-based parental haplotyping
Source: Genome Med. 2021 Feb 5;13:18. doi: 10.1186/s13073-021-00836-8 (PMC7866698; doi:10.1186/s13073-021-00836-8)
Supplement: Supplementary file 6 — Additional file 6: Table S3. Concordance of parental haplotypes deduced by PBH and FBH. [file 13073_2021_836_MOESM6_ESM.docx]

**Additional file 6: Table S3 Concordance of parental haplotypes deduced by PBH and FBH.**

| **Family** | **No. of SNPs in the Mother** | | | |  | **No. of SNPs in the Father** | | | |
| --- | --- | --- | --- | --- | --- | --- | --- | --- | --- |
|  | **No. of SNPs Phased by PBH ^a^** | **No. of SNPs Phased by PBH and FBH ^b^** | **No. of Consistent SNPs ^c^** | **Concordance Rate ^d^** |  | **No. of SNPs Phased by PBH ^a^** | **No. of SNPs Phased by PBH and FBH ^b^** | **No. of Consistent SNPs ^c^** | **Concordance Rate ^d^** |
| F01 | 81 | 66 | 66 | 100% |  | 90 | 75 | 75 | 100% |
| F02 | 127 | 105 | 105 | 100% |  | 124 | 102 | 101 | 99.0% |
| F03 | 56 | 56 | 56 | 100% |  | 71 | 71 | 42 | 59.2% |
| F04 | 104 | 74 | 72 | 97.3% |  | 161 | 123 | 123 | 100% |
| F05 | 87 | 55 | 53 | 96.4% |  | 144 | 112 | 111 | 99.1% |
| F06 | 45 | 14 | 14 | 100% |  | 126 | 95 | 95 | 100% |
| F07 | 116 | 114 | 114 | 100% |  | 27 | 25 | 25 | 100% |
| F08 | 81 | 60 | 58 | 96.7% |  | 106 | 85 | 51 | 60.0% |
| F09 | 13 | 7 | 7 | 100% |  | 81 | 75 | 75 | 100% |
| F10 | 36 | 15 | 15 | 100% |  | 48 | 27 | 27 | 100% |
| F11 | 51 | 33 | 33 | 100% |  | 109 | 91 | 54 | 59.3% |
| F12 | 115 | 104 | 104 | 100% |  | 35 | 24 | 24 | 100% |
| F13 | 178 | 178 | 178 | 100% |  | 29 | 29 | 27 | 93.1% |
| F14 | 66 | 60 | 60 | 100% |  | 97 | 91 | 91 | 100% |
| F15 | 189 | 164 | 162 | 98.8% |  | 55 | 30 | 30 | 100% |
| F16 | 94 | 67 | 67 | 100% |  | 83 | 56 | 37 | 66.1% |
| F17 | 123 | 112 | 112 | 100% |  | 99 | 88 | 75 | 85.2% |

**Additional file 6: Table S3** **Concordance of parental haplotypes deduced by PBH and FBH (continued)**

| **Family** | **No. of SNPs in the Mother** | | | |  | **No. of SNPs in the Father** | | | |
| --- | --- | --- | --- | --- | --- | --- | --- | --- | --- |
|  | **No. of SNPs Phased by PBH ^a^** | **No. of SNPs Phased by PBH and FBH ^b^** | **No. of Consistent SNPs ^c^** | **Concordance Rate ^d^** |  | **No. of SNPs Phased by PBH ^a^** | **No. of SNPs Phased by PBH and FBH ^b^** | **No. of Consistent SNPs ^c^** | **Concordance Rate ^d^** |
| F18 | 102 | 81 | 81 | 100% |  | 75 | 54 | 51 | 94.4% |
| F19 | 119 | 112 | 110 | 98.2% |  | 49 | 42 | 41 | 97.6% |
| F20 | 107 | 107 | 107 | 100% |  | 48 | 48 | 48 | 100% |
| F21 | 75 | 68 | 68 | 100% |  | 86 | 79 | 77 | 97.5% |
| F22 | 47 | 42 | 40 | 95.2% |  | 50 | 45 | 45 | 100% |
| F23 | 78 | 78 | 78 | 100% |  | 72 | 72 | 59 | 81.9% |
| F24 | 67 | 32 | 28 | 87.5% |  | 74 | 39 | 39 | 100% |
| F25 | 44 | 40 | 40 | 100% |  | 83 | 79 | 79 | 100% |
| F26 | 93 | 48 | 48 | 100% |  | 80 | 35 | 35 | 100% |
| F27 | 24 | 9 | 8 | 88.9% |  | 46 | 31 | 31 | 100% |
| F28 | 75 | 37 | 37 | 100% |  | 68 | 30 | 30 | 100% |
| F29 | 99 | 97 | 96 | 99.0% |  | 103 | 101 | 100 | 99.0% |
| F30 | 77 | 74 | 74 | 100% |  | 63 | 60 | 60 | 100% |
| F31 | 88 | 85 | 83 | 97.6% |  | 52 | 50 | 49 | 98.0% |
| F32 | 70 | 44 | 42 | 95.5% |  | 75 | 49 | 49 | 100% |
| F33 | 49 | 19 | 19 | 100% |  | 111 | 81 | 81 | 100% |
| F34 | 60 | 15 | 15 | 100% |  | 64 | 19 | 19 | 100% |

**Additional file 6: Table S3** **Concordance of parental haplotypes deduced by PBH and FBH (continued)**

| **Family** | **No. of SNPs in the Mother** | | | |  | **No. of SNPs in the Father** | | | |  |
| --- | --- | --- | --- | --- | --- | --- | --- | --- | --- | --- |
|  | **No. of SNPs Phased by PBH ^a^** | **No. of SNPs Phased by PBH and FBH ^b^** | **No. of Consistent SNPs ^c^** | **Concordance Rate ^d^** |  | **No. of SNPs Phased by PBH ^a^** | **No. of SNPs Phased by PBH and FBH ^b^** | **No. of Consistent SNPs ^c^** | **Concordance Rate ^d^** | |
| F35 | 72 | 48 | 48 | 100% |  | 55 | 31 | 31 | 100% | |
| F36 | 83 | 57 | 57 | 100% |  | 48 | 22 | 22 | 100% | |
| F37 | 32 | 31 | 31 | 100% |  | 40 | 39 | 39 | 100% | |
| F38 | 101 | 99 | 99 | 100% |  | 50 | 48 | 48 | 100% | |
| F39 | 57 | 45 | 44 | 97.8% |  | 59 | 47 | 47 | 100% | |
| F40 | 65 | 27 | 27 | 100% |  | 47 | 9 | 9 | 100% | |
| F41 | 76 | 28 | 28 | 100% |  | 100 | 53 | 51 | 96.2% | |
| F42 | 65 | 43 | 43 | 100% |  | 67 | 45 | 45 | 100% | |
| F43 | 101 | 62 | 61 | 98.4% |  | 57 | 18 | 17 | 94.4% | |
| F44 | 57 | 54 | 54 | 100% |  | 103 | 100 | 100 | 100% | |
| F45 | 106 | 49 | 49 | 100% |  | 78 | 21 | 21 | 100% | |
| F46 | 60 | 53 | 52 | 98.1% |  | 32 | 25 | 21 | 84.0% | |
| F47 | 98 | 8 | 8 | 100% |  | 98 | 8 | 8 | 100% | |
| F48 | 66 | 64 | 63 | 98.4% |  | 110 | 108 | 108 | 100% | |
| F49 | 46 | 42 | 42 | 100% |  | 48 | 44 | 43 | 97.7% | |
| F50 | 103 | 100 | 99 | 99.0% |  | 60 | 56 | 56 | 100% | |
| F51 | 67 | 17 | 17 | 100% |  | 102 | 52 | 51 | 98.1% | |

**Additional file 6: Table S3** **Concordance of parental haplotypes deduced by PBH and FBH (continued)**

| **Family** | **No. of SNPs in the Mother** | | | | |  | **No. of SNPs in the Father** | | | |
| --- | --- | --- | --- | --- | --- | --- | --- | --- | --- | --- |
|  | **No. of SNPs Phased by PBH ^a^** | | **No. of SNPs Phased by PBH and FBH ^b^** | **No. of Consistent SNPs ^c^** | **Concordance Rate ^d^** |  | **No. of SNPs Phased by PBH ^a^** | **No. of SNPs Phased by PBH and FBH ^b^** | **No. of Consistent SNPs ^c^** | **Concordance Rate ^d^** |
| F52 | 80 | 25 | | 24 | 96.0% |  | 97 | 42 | 42 | 100% |
| F53 | 6 | 4 | | 4 | 100% |  | 49 | 47 | 47 | 100% |
| F54 | 69 | 63 | | 60 | 95.2% |  | 86 | 82 | 82 | 100% |
| F55 | 101 | 97 | | 97 | 100% |  | 59 | 56 | 56 | 100% |
| F56 | 56 | 45 | | 45 | 100% |  | 59 | 48 | 48 | 100% |
| F57 | 65 | 61 | | 61 | 100% |  | 15 | 11 | 10 | 90.9% |
| F58 | 78 | 77 | | 71 | 92.2% |  | 2 | 1 | 1 | 100% |
| F59 | 62 | 59 | | 58 | 98.3% |  | 47 | 44 | 43 | 97.7% |

^a^ The number of phased SNPs in parents inferred by PBH. ^b^ The number of phased SNPs in parents inferred by two methods (PBH and FBH). ^c^ The number of phased SNPs that were consistent between the two methods. ^d^ Concordance rate = c/b. Note that only part of the phased SNPs listed in the column (No. of SNPs Phased by PBH ^a^) can satisfy the criteria of informative SNPs (described in the Additional file 3) to infer fetal haplotypes; thus, the number of informative SNPs in Table 1 is lower than that in the column (No. of SNPs Phased by PBH ^a^).
