# Supplementary material for: Noninvasive prenatal testing of α-thalassemia and β-thalassemia through population-based parental haplotyping
Source: Genome Med. 2021 Feb 5;13:18. doi: 10.1186/s13073-021-00836-8 (PMC7866698; doi:10.1186/s13073-021-00836-8)
Supplement: Supplementary file 7 — Additional file 7: Fig. S3. NIPT results for thalassemia. [file 13073_2021_836_MOESM7_ESM.docx]

**
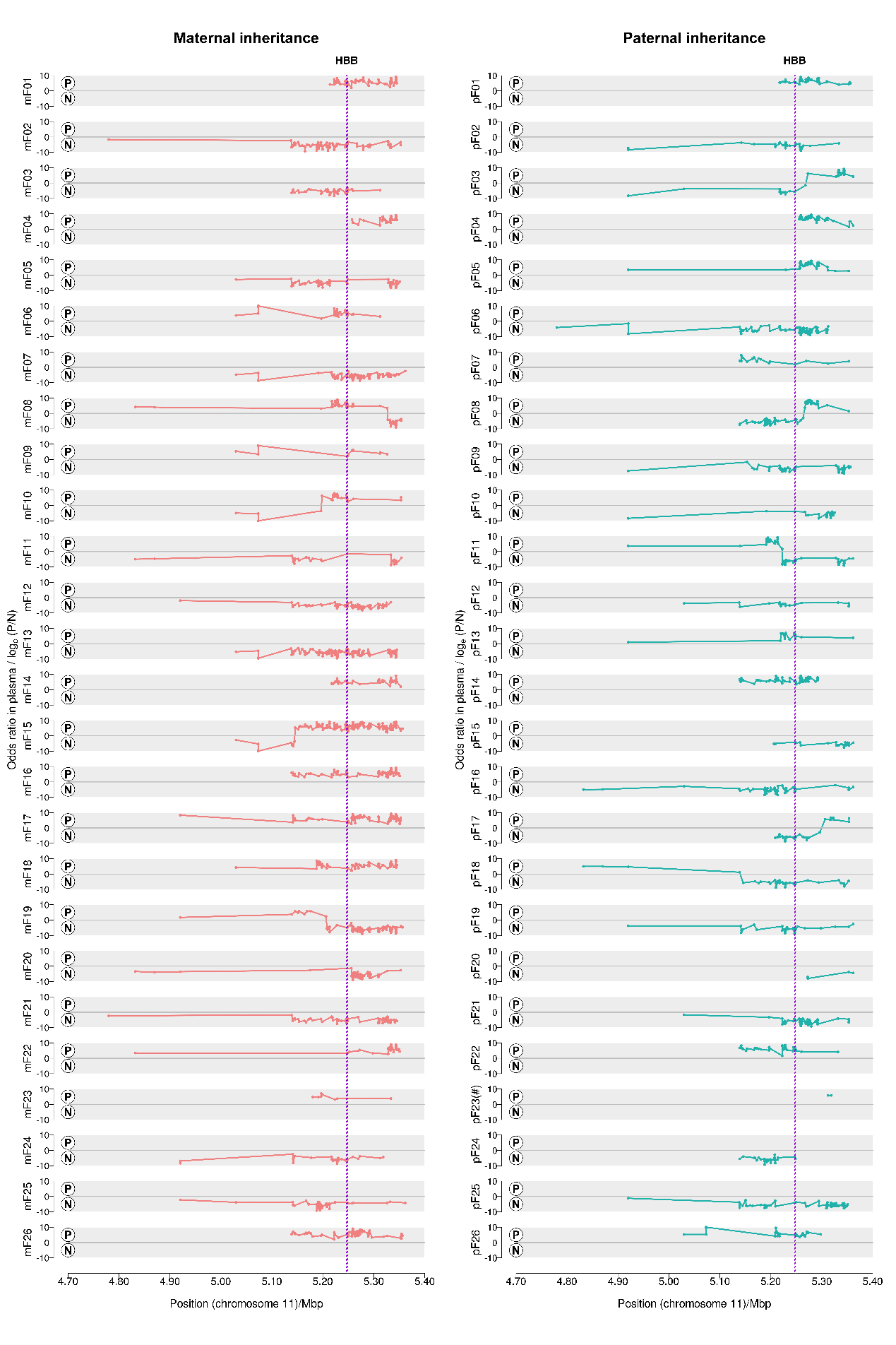
Figure S3a**


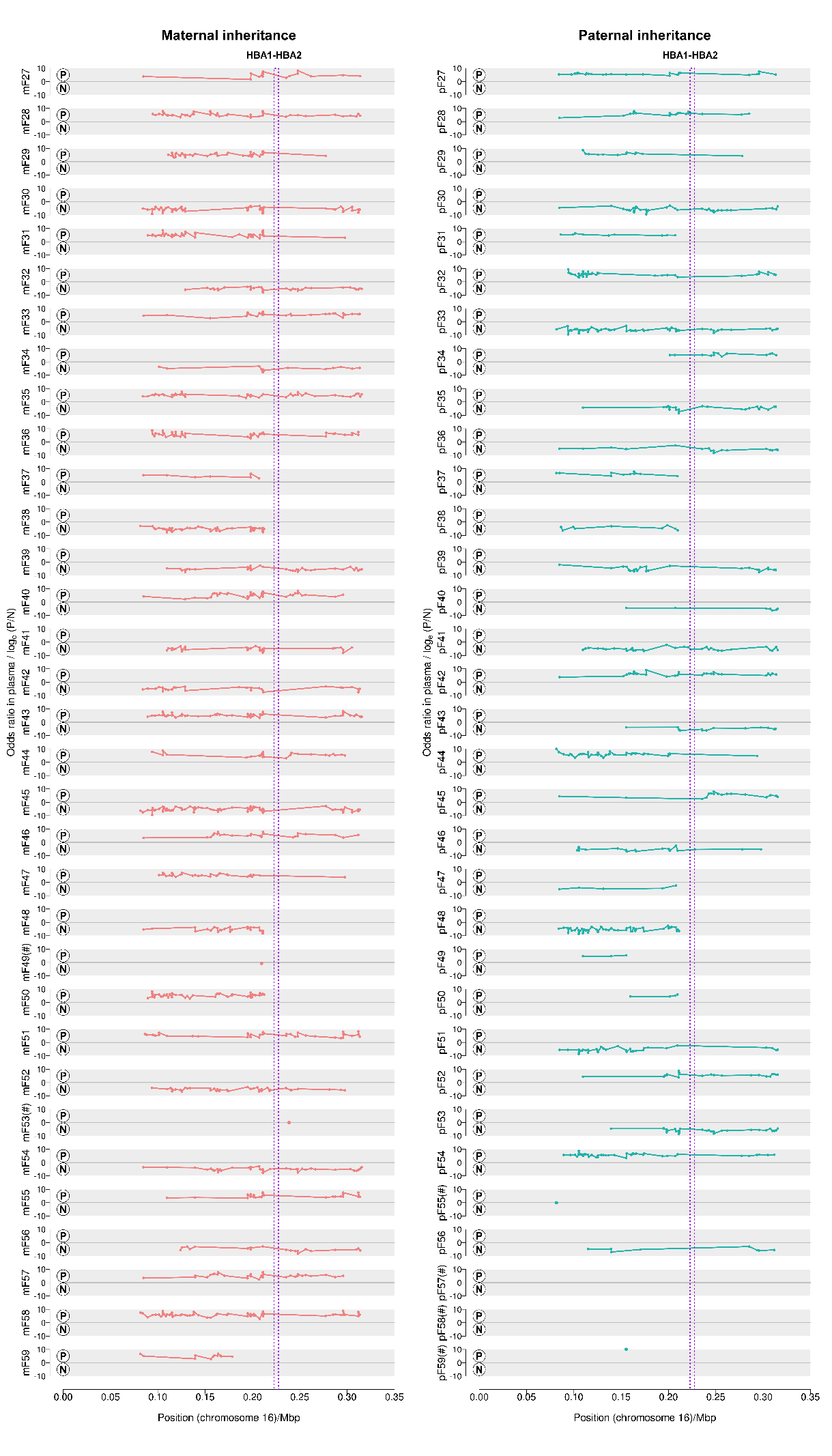
**Figure S3b**

**Additional file 7: Figure S3. NIPT** **results for thalassemia.**

The x-axis indicates the genomic coordinates of the target region (Mbp), the y-axis indicates the log_e_ of the odds ratio of the pathogenicity for each informative SNP, and each red/green line connecting consecutive SNPs (represented as dots) indicates a maternal/paternal haplotype transmission path to the fetus. While the paths above zero (gray lines) indicate inheritance of a pathogenic haplotype (**P**), the paths below zero indicate inheritance of a normal haplotype (**N**). The purple vertical lines indicate the target genes. Fetal inheritance conditions determined to be no-call are marked with (#). **a,** NIPT results for β-thalassemia. **b,** NIPT results for α-thalassemia.
