# Supplementary material for: Noninvasive prenatal testing of α-thalassemia and β-thalassemia through population-based parental haplotyping
Source: Genome Med. 2021 Feb 5;13:18. doi: 10.1186/s13073-021-00836-8 (PMC7866698; doi:10.1186/s13073-021-00836-8)
Supplement: Supplementary file 8 — Additional file 8: Table S4. Cost and turnaround time of PBH-NIPT. [file 13073_2021_836_MOESM8_ESM.docx]

**Additional file 8: Table S4 Cost and turnaround time of PBH-NIPT**

|  | **Estimated cost**  **per sample** | **Turnaround time** |
| --- | --- | --- |
| Cell-free DNA extraction and QC | ~**$**5 | ~0.5 day |
| Library preparation and QC | ~**$**15 | ~0.5 day |
| Hybridization capture and QC (8 indexed libraries pooled into one sequencing library) | ~**$**30 | ~2 days |
| Sequencing | ~**$**25 | ~2 days |
| Data analysis | ~**$**5 | ~1-2 days |
| **Total** | **~$80** | **~7 days** |
